# Contactless steam generation and superheating under one sun illumination

**DOI:** 10.1038/s41467-018-07494-2

**Published:** 2018-12-11

**Authors:** Thomas A. Cooper, Seyed H. Zandavi, George W. Ni, Yoichiro Tsurimaki, Yi Huang, Svetlana V. Boriskina, Gang Chen

**Affiliations:** 1 Department of Mechanical Engineering, Massachusetts Institute of Technology, 77 Massachusetts Ave., Cambridge, Massachusetts 02139 USA; 2 Present Address: Department of Mechanical Engineering Lassonde School of Engineering, York University, 4700 Keele St., Toronto, Ontario M3J 1P3 Canada

## Abstract

Steam generation using solar energy provides the basis for many sustainable desalination, sanitization, and process heating technologies. Recently, interest has arisen for low-cost floating structures that absorb solar radiation and transfer energy to water via thermal conduction, driving evaporation. However, contact between water and the structure leads to fouling and pins the vapour temperature near the boiling point. Here we demonstrate solar-driven evaporation using a structure not in contact with water. The structure absorbs solar radiation and re-radiates infrared photons, which are directly absorbed by the water within a sub-100 μm penetration depth. Due to the physical separation from the water, fouling is entirely avoided. Due to the thermal separation, the structure is no longer pinned at the boiling point, and is used to superheat the generated steam. We generate steam with temperatures up to 133 °C, demonstrating superheated steam in a non-pressurized system under one sun illumination.

## Abstract (web summary)

Solar steam generation is limited by fouling of solar converters, and the steam temperature is usually pinned to 100 °C. Here, both limitations are overcome in a system utilizing a solar absorber and light down-converter to achieve radiative heating, which does not require physical contact between absorber and water.

## Introduction

The sun constitutes a vast yet largely untapped source of clean and renewable energy. The potential of solar energy is not limited to direct conversion to electricity: solar energy can be transformed into other useful forms, notably heat, which can then be used to drive industrial processes^1^, provide residential space heating^2^ or cooling^3^ or to displace fossil fuels as the heat source in conventional power plants^4^. In particular, solar-driven desalination^5^ provides one potential answer to the grand challenge of providing low-cost clean drinking water to the planet. Recent work has generated a variety of low-cost solar absorbers that can be deployed directly in bodies of water to generate vapour^6–19^. Absorber materials range from black paints and fabrics historically used in solar stills^20,21^, to nanoparticle suspensions^13,22–24^, high-porosity membranes^6,15,25,26^ and nano-patterned materials^27^. Regardless of the material used, a common feature of previous approaches is the requirement of physical contact between the material and the liquid water to affect heat transfer by thermal conduction. However, many solar vapour applications rely on contaminated water sources. During evaporation, vapour leaves the water source, leaving behind concentrated salts and impurities that contaminate and clog the absorbing structures, an issue collectively referred to as fouling. Attempts to address the fouling issue have included daily cleaning and rinsing, material recycling^23^ and, more recently, development of anti-fouling and salt-rejecting structures^28,29^. While significant strides have been made, fouling remains a fundamental challenge inherent to all solar absorbers in direct contact with the water surface.

Additionally, as long as the absorber remains in contact with water, the achievable vapour temperature is pinned near the boiling point of water (100 °C at atmospheric pressure). Oftentimes, applications demand higher temperatures, for example, sterilization, where health safety standards require steam at 121–135 °C to kill pathogenic microorganisms and their spores^30^. Prior to this work, access to such temperatures normally necessitated pressurization^31^ to achieve boiling-point elevation. The innovative structure developed by Zhang et al.^32^ achieved temperatures as high as 121 °C without pressurization, but required solar concentration ratios in excess of 20 suns (1 sun = 1000 W m^−2^) to achieve this.

In this work, we demonstrate a solar evaporation structure, wherein the solar absorber does not touch the water surface. In this contactless configuration, energy is transferred to the water via thermal radiation, a non-contact mode of heat transfer, thus circumventing the absorber fouling problem. Unlike sunlight, mid- and far-infrared thermal radiation is very efficiently absorbed directly by the water, which thereby serves as its own absorber. In addition to physically decoupling the solar evaporation structure from the water, the contactless configuration thermally decouples the absorber structure from the boiling point of water. With the structure no longer pinned at the boiling point, additional heat can be transferred from the structure to the generated steam, thus bringing it out of the saturated state into the superheated state. Through superheating, steam temperatures well in excess of 100 °C are achieved under one-sun conditions, without the need for pressurization. In this light, the contactless configuration both addresses the fouling issue and boosts the achievable steam temperature, thus opening the door to new applications, including sterilization, autoclaving, cooking and medium temperature that processes heat.

## Results

### Contactless solar steam generation via thermal downconversion

**Fig. 1:**
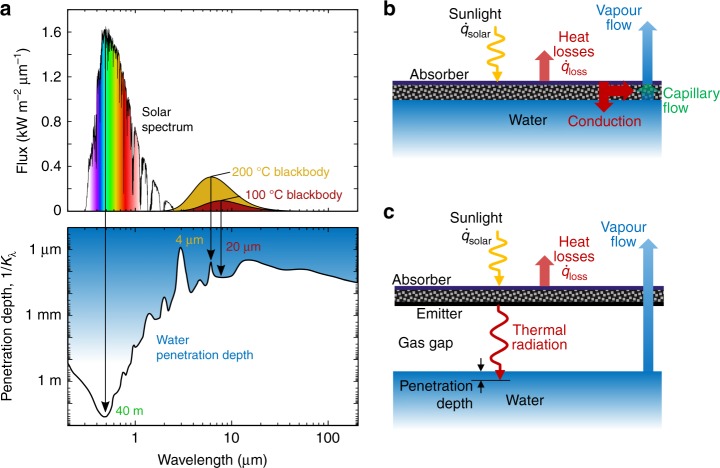
Operating principle of contactless solar evaporation via thermal downconversion. **a** Bottom panel: photon penetration depth (reciprocal of absorption coefficient) for liquid water. Top panel: flux spectrum for solar radiation and for thermal blackbody sources at 100 and 200 °C. The penetration depth for solar photons is several orders of magnitude higher than that for thermal infrared photons. **b** In a conventional solar evaporation structure generator, a solar absorber is placed in contact with the water, and transfers heat from the absorbed sunlight to the water via thermal conduction. **c** In the proposed contactless solar evaporation structure, the absorber is not in contact with the water. As the absorber heats up, it emits thermal radiation to the water, which is absorbed within a very thin layer (<100 μm) beneath the water/vapour interface

Fundamentally, solar steam generation is a process by which solar energy is used to drive the endothermic phase transition from liquid water to vapour. A necessary step for this process is the energy transfer from solar photons to the water molecules. Unfortunately, water is a poor absorber of photons at solar wavelengths. The absorption of a beam of radiation as it propagates through an absorbing medium is described by the Beer–Lambert law

1 $$\tau _\lambda (L) = I_\lambda (L)/I_{\lambda ,0} = e^{ - \kappa _\lambda L}$$

where *τ*_λ_(*L*) is the spectral transmittance, defined as the intensity *I*_*λ*_ of a beam at a distance *L*, relative to the incident intensity *I*_*λ*,0_ at *L* = 0 (see Supplementary Note 1 for details of the nomenclature). The spectral absorption coefficient the *κ*_λ_ quantifies the strength of absorption in the water, and its 1/*κ*_λ_ can be interpreted as the penetration depth of a photon of wavelength *λ*. The bottom panel of Fig. 1a shows the penetration depth spectrum for water, computed from its optical constants^33^. Comparison with the solar spectrum^34^ (top panel of Fig. 1a) reveals that the peak solar irradiance coincides with the maximum water penetration depth, which exceeds 40 m. Therefore, impractically large depths are required to directly absorb solar radiation using water. By spectrally averaging (see Supplementary Note 2), the depth required to absorb 90% of the incident solar radiation is found to be greater than 20 m (see Supplementary Fig. 1). Previous solar evaporation structure designs, shown schematically in Fig. 1b, have overcome this absorption problem through use of an intermediate optical absorber, which may be a monolithic structure^7^, or a suspension of absorbing particles distributed through the water^13,35,36^. A common feature of existing systems is that the absorber is in contact with the water and transfers the absorbed solar energy to the water via thermal conduction. Even in the case when an insulating layer is placed between the absorber and water layer^7^, previous systems have relied on wicking via capillary action^6^ or gravity^37^ to bring the water in contact with the absorber such that heat can be transferred by thermal conduction.

In contrast to solar wavelengths, photons at infrared wavelengths are readily absorbed by liquid water. Superimposed in Fig. 1a are the irradiance spectra for blackbody sources at 100 and 200 °C. The thermal spectra span a wavelength range which coincides with the strong vibrational absorption bands of the H_2_O molecule^38^, where the penetration depths range from 1 to 100 μm, many orders of magnitude smaller than for solar wavelengths. For a blackbody source below 500 °C, the depth required to absorb 90% of the incident energy is less than 100 μm (Supplementary Fig. 1), far below the 20 m needed for solar radiation.

We leverage the noteworthy infrared absorption properties of water to enable a contactless solar evaporation structure (Fig. 1c). Rather than placing a solar-absorbing material in contact with the water, we instead modify the solar spectrum by down-converting it to thermal infrared radiation which is then directly absorbed by the water. Incident solar energy is converted to heat by the absorber, and consequently re-emitted as thermal radiation towards the water, which approaches blackbody behaviour in the thermal wavelengths (see Supplementary Fig. 2). Owing to the excellent infrared absorption properties of water, this emitted radiation is absorbed in a sub-100-μm layer at the top of the water, effectively allowing the water to serve as its own absorber.

### Achieving superheated steam at low solar flux

In the conventional scheme (Fig. 1b), the absorber is in close thermal contact with the water and its temperature is limited to the boiling point, *T*_b_, which equals 100 °C at an ambient pressure of 1 atm. In contrast, the contactless arrangement (Fig. 1c) physically and thermally separates the absorber/emitter from the water. Consequently, the absorber temperature *T*_e_ is not pinned to *T*_b_. Consider a simplified 1D system like that shown in Fig. 1c. A steady-state energy balance on the absorber/emitter gives the net heat flux transferred to the water as *q̇*_gain_ = *η*_opt_·*q̇*_solar_ − *q̇*_loss_, where *η*_opt_ is the optical efficiency, *q̇*_solar_ is the incident solar flux, and *q̇*_loss_ represents all forms of heat loss to the environment (see Supplementary Fig. 3). By defining overall heat transfer coefficients: *U*_gain_ ≡ *q̇*_gain_/(*T*_e_ − *T*_w_) and *U*_loss_ ≡ *q̇*_loss_/(*T*_e_ − *T*_∞_), where *T*_w_ and *T*_∞_ are the water and ambient temperatures, respectively, we can solve the energy balance for the steady-state emitter temperature (see Supplementary Note 3).

2 $$T_{\mathrm{e}} = \frac{{\eta _{{\mathrm{opt}}}\dot q_{{\mathrm{solar}}} + U_{{\mathrm{gain}}}T_{\mathrm{w}} + U_{{\mathrm{loss}}}T_\infty }}{{U_{{\mathrm{loss}}} + U_{{\mathrm{gain}}}}}$$

As representative conditions, we take *q̇*_solar_ = 1000 W m^−2^, *η*_opt_ = 0.76 (see Supplementary Note 4), and *T*_w_ = 100 °C. In general, *U*_gain_ and *U*_loss_ are dependent on *T*_e_, such that Eq. (2) must be solved iteratively. However, a simple explicit model is be obtained by assuming constant representative values for *U*_gain_ and *U*_loss_. For illustrative purposes, we take *U*_loss_ = 4.6 W m^−2^K^−1^, which is approximately what was achieved in our demonstration (see Supplementary Table 1), and encompasses radiation, conduction and convection heat losses from the system to environment. For an estimate of *U*_gain_, we assume blackbody exchange between a planar emitter and water, such that *U*_gain_ is the radiation heat transfer coefficient $\sigma (T_{\mathrm{w}}^2 + T_{\mathrm{e}}^2)(T_{\mathrm{w}} + T_{\mathrm{e}})$ which amounts to 13 W m^−2^K^−1^ for *T*_w_ = 373 K and *T*_e_ = 398 K. Equation (2) can then be solved explicitly to give an equilibrium emitter temperature of 124 °C. The importance of this high emitter temperature is the potential to superheat the generated steam to temperatures above 100 °C. Figure 1c shows the steam superheating mechanism. Before escaping to the environment, the initially produced 100 °C saturated steam is forced to follow a tortuous path through the emitter. The hot emitter transfers heat to the steam bringing it into the superheated state (e.g., vapour whose temperature is above the saturation temperature for a given pressure), maximally superheating it to *T*_e_. In this simple analytical model, the sensible heat of the steam has been neglected in the energy balance on the absorber/emitter, since it is small in comparison with the other energy terms (Supplementary Note 3). To complement the simple analytical model described here (and detailed in Supplementary Note 3), we have developed a detailed transient numerical heat transfer model (see Supplementary Note 5), which models the system using a distributed thermal circuit (see Supplementary Fig. 4), captures nonlinear effects (for example the radiative exchange between the emitter and the water, see Supplementary Fig. 5), and includes the sensible heat of superheating.

### Laboratory-scale contactless solar evaporation structure

**Fig. 2:**
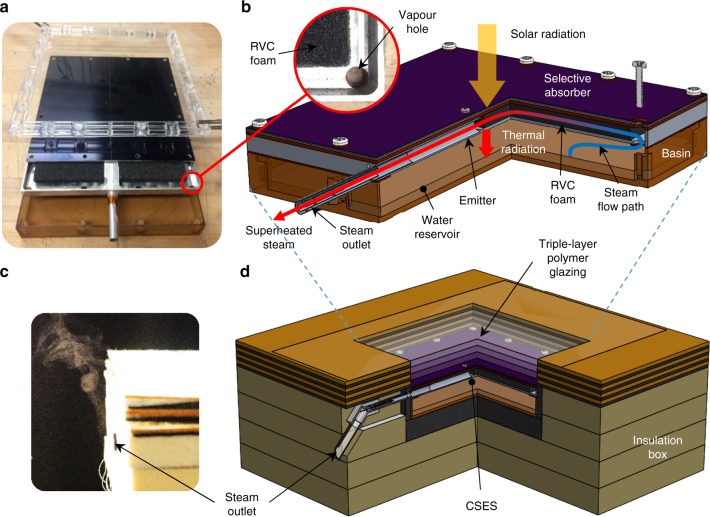
Laboratory-scale contactless solar evaporation structure (CSES). **a** Photograph of disassembled CSES device. **b** Cutaway schematic of CSES device showing the main components and steam flow path. **c** Photograph of steam generated by the CSES at one sun illumination. **d** Cutaway schematic of the laboratory experiment

We developed a laboratory-scale contactless solar evaporation structure (CSES) to demonstrate both non-contact evaporation and steam superheating at low solar flux levels. Figure 2a shows a photograph of the disassembled CSES device, and Fig. 2b gives a cutaway schematic indicating its most important components. Details of the design are presented in Supplementary Note 6 and Supplementary Fig. 6. The CSES was placed in an insulated enclosure (see Supplementary Fig. 7) and operated under simulated solar radiation at solar flux levels ranging from 0.5 to 1.8 suns. During the lab-scale demonstration, we successfully produced superheated steam, which could be directly observed during the experiment (see Fig. 2c and Supplementary Movie). The simple steady-state analytical model (Supplementary Note 3) and detailed transient numerical model (Supplementary Note 5) were validated vis-à-vis the experiments, and subsequently used to gain insight into the evaporation mechanism and to guide future CSES design optimization.

**Fig. 3:**
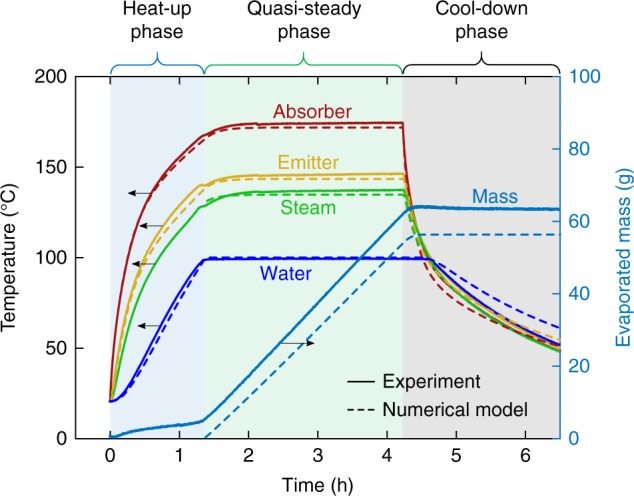
Transient response of the CSES under 1.5 sun illumination. Experimentally measured (solid lines) and modelled (dashed lines) transient response of the absorber, emitter, water and steam temperatures for a representative experimental run at 1.5 suns. Also shown is the temporal evolution of the evaporated mass measured by a balance, and predicted by the transient model (Supplementary Note 5), which accurately predicts the mass flux during the quasi-steady phase. The small initial evaporated mass during the heat-up phase is partially attributed to evaporation of residual moisture in the porous materials, and is not accounted for the in the model which predicts the start of evaporation when *T*_w_ reaches 100 °C

The function of the CSES can be best understood by following the transient response of the system over the course of an experiment. Figure 3 shows the temperature evolution for a representative laboratory-scale run at 1.5 suns. The experiment exhibits three distinct phases: an initial heat-up phase (illuminated), a quasi-steady-state phase (illuminated) and a cool-down phase (dark).

During the heat-up phase, sunlight incident on the device causes it to heat up at a rate of around 2 K/min. Radiation heat losses to the environment are minimized by a spectrally selective absorber material, which has a high solar absorptance *α*_solar_ of 0.92 and a low thermal emittance *ϵ*_thermal_ of 0.08 (Supplementary Fig. 8). Convective losses to the environment are minimized by a three-layer transparent polymer glazing system shown in Fig. 2d. The glazing achieves an effective heat transfer coefficient of 1.66 W m^−2^ K^−1^, while maintaining a high solar transmittance *τ*_solar_ of 0.86 (see Supplementary Fig. 9). Heat is conducted from the absorber to the emitter, which comprises an aluminium shell whose bottom surface is painted with a high thermal emittance coating (*ϵ*_thermal_ = 0.94). Details of optical and radiative property measurements are provided in Supplementary Note 7, and key properties are summarized in Supplementary Table 2. As the emitter heats up, it radiates directly to the water reservoir below. This thermal radiation is absorbed in a thin layer (~30 μm) at the top of the water surface causing it to heat up at a rate of ~1 K/min. This is relatively slow compared with the heating rates in heat localizing structures^7^, which achieve above 10 K/min at one sun, primarily due to the comparatively high thermal mass of the CSES and water reservoir. Faster heat up rates could be achieved by specifically optimizing the CSES for a lower thermal mass, or simply by reducing the initial depth of the water layer in the reservoir at the start of the experiment (see Supplementary Note 8 and Supplementary Fig. 10). Efficient operation of the CSES on deep water basins could be facilitated by the addition of a perforated layer of thermal insulation, submerged a few millimetres below the water surface, thus localizing the heat to a thin water layer^6^. Such a device could be designed to float on open pools with the desired submerged depth of insulation passively controlled by buoyancy.

Heat transfer from the emitter to the water surface occurs predominantly by radiation, with small contributions from thermal conduction through the gas gap and the basin sidewalls. Under representative conditions, the detailed model predicts 82% via radiation, 12% via gas gap thermal conduction, and 6% via sidewall conduction. Interestingly, the upward flow induced by evaporation actually reduces the conduction heat transfer in the gas gap compared with pure conduction which would occur for a stationary gas gap (see Supplementary Note 9 and Supplementary Fig. 11). Due to the low Biot number (see Supplementary Note 5), the water reservoir is heated uniformly, and can be treated as approximately isothermal through the course of the experiment, even though the radiation is absorbed in a thin layer below the surface.

When the water reaches 100 °C, its temperature plateaus, marking the start of the quasi-steady-state phase. With the water at its boiling point, each additional unit of absorbed energy goes towards evaporation at the water/vapour interface, and a sharp rise in the evaporated mass curve is observed (Fig. 3). The evaporation rate is heat transfer controlled, and the mass flux *j* (evaporation mass flow rate per unit absorber area) during the quasi-steady phase can be directly determined from the heat flux according to

3 $$j = \dot q_{{\mathrm{gain}}}/h_{{\mathrm{fg}}}$$

where *h*_fg_ = 2257 kJ/kg is the latent heat of vaporization of water at 100 °C. During the quasi-steady phase, the modelled evaporated mass curve seen in Fig. 3, closely matches the experimental curve, which was measured continuously using a balance (see Methods). For the experiment shown in Fig. 3, a steady-state mass flux of 0.26 g s^−1^ m^−2^ was measured.

Generated vapour leaves the water surface as saturated steam at *T*_b_ and rises towards the emitter, where it enters the superheater through 12 vapour holes in the emitter surface (see close-up in Fig. 2b), and then flows laterally in between the absorber and emitter towards the central steam outlet tube. To promote good solid–vapour heat exchange, we sandwiched a highly porous reticulated vitreous carbon (RVC) foam between the absorber and emitter. As the steam flows through the RVC, heat is transferred from the hot absorber/emitter to the steam, bringing it into the superheated state. The lateral flow arrangement forces the steam through a long tortuous path, promoting solid–vapour heat transfer, thus maximizing the degree of superheating. For the experiment shown in Fig. 3 (1.5 suns), a peak steam temperature of 135 °C was measured. The superheated steam is forced through a single-outlet tube and is vented directly to the atmosphere (Fig. 2c). A custom-built radiation-shielded thermocouple (see Supplementary Note 10 and Supplementary Fig. 12) was mounted in the centre of the outlet tube to accurately measure the superheated steam temperature. Since the steam temperature is measured accurately only when there is significant vapour flow (see Supplementary Note 10), which occurs when the water reaches 100 °C, the steam temperature curve should be interpreted with caution during the heat-up phase. At the end of the steady-state phase the solar simulator is turned off and the device begins to slowly cool back to the ambient temperature.

### Efficiency

**Fig. 4:**
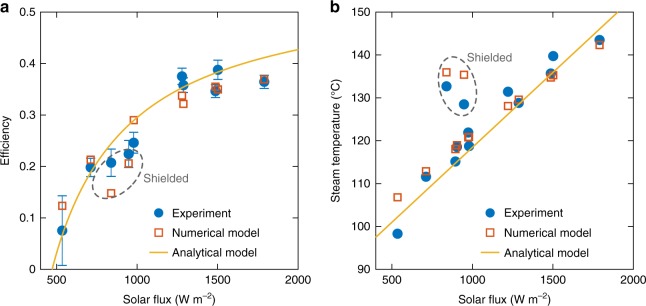
Performance of a laboratory-scale contactless solar evaporation structure. **a** Measured and modelled steady-state efficiency as a function of the incident solar flux. Each data point corresponds to a single unique experimental run. The steady-state efficiency was determined by dividing the evaporated mass during the quasi-steady phase Δ*m*_ss_ by the duration of the quasi-steady phase Δ*t*_ss_. The evaporated mass was determined by measuring change in the mass of water in the basin before and after the experiment Δ*m*_basin_. Error bars indicate uncertainty in steady-state efficiency due to the small amount of evaporation in the heat-up phase (see Fig. 3) Δ*m*_heat-up_, which is attributed partially to evaporation from the basin, and partially to evaporation of residual moisture in the system. For the lower bound, Δ*m*_heat-up_ is attributed solely to basin evaporation, is thus subtracted from Δ*m*_basin_ to obtain a rigorous lower bound on efficiency. For the upper bound, Δ*m*_heat-up_ is attributed entirely to residual moisture and thus does not need to be subtracted from Δ*m*_basin_. The marker location indicates a representative intermediate value where we attribute 50% of Δ*m*_heat-up_ as being due to basin evaporation (see Supplementary Note 11 for details). **b** Measured and modelled superheated steam temperature during the quasi-steady region as a function of the incident solar flux. The analytical model is described in Supplementary Note 3, and is summarized by Eqs. (5) and (6). The numerical model is described in Supplementary Note 5. For the results labelled “shielded”, a radiation shield was used to boost the steam temperature

The overall performance of a solar evaporation structure is quantified by its solar to thermal conversion efficiency, defined as^7^

4 $$\eta = j \cdot h_{{\mathrm{fg}}}/\dot q_{{\mathrm{solar}}}$$

where *q̇*_solar_ is the incident solar flux. The lab-scale CSES achieved an efficiency of 24.6% at one sun conditions, and a maximum value of 38.8% at 1.5 suns. This definition does not include the sensible heat of superheating in the numerator allowing direct comparison with previous investigations. Under representative conditions, the efficiency including sensible heat is ~1 absolute percentage point higher than that given by Eq. (4) (see Supplementary Note 3). Figure 4a shows the measured efficiency during the steady-state region as a function of the solar flux (see Methods and Supplementary Note 11 for details of the efficiency calculation). The contactless configuration operates at lower efficiencies than contact evaporation methods and traditional solar stills^39^, due to the higher absorber and steam temperatures. However, an additional advantage of the CSES design is the built-in vapour collection, since steam is delivered to a single outlet tube. Previous works generated vapour distributed over a wide area, requiring collection by a semi-transparent condensing cover^21,40,41^. When losses due to the condensing cover are taken into account, the CSES performance is comparable with the lower-temperature evaporation systems that we have investigated^28^, which achieve efficiencies in the range 21–24% with collection.

As shown in Supplementary Note 3, the analytical model gives a simple expression for the efficiency as

5 $$\eta = \frac{{U_{{\mathrm{gain}}}}}{{U_{{\mathrm{loss}}} + U_{{\mathrm{gain}}}}}\left( {\eta _{{\mathrm{opt}}} - U_{{\mathrm{loss}}}\frac{{T_{\mathrm{w}} - T_\infty }}{{\dot q_{{\mathrm{solar}}}}}} \right)$$

*U*_gain_ and *U*_loss_ can either be predicted from detailed heat transfer analysis of the device, or can be fit to experimental data. Superimposed in Fig. 4a are the predictions of Eq. (5) for best-fit parameters *U*_loss_ = 4.6 W m^−2^K^−1^, *U*_gain_ = 12.8 W m^−2^K^−1^, and *η*_opt_ = 75.8% (c.f. Supplementary Table 1). Also superimposed are the predictions of the detailed numerical model, with both models showing good agreement with the experimental results.

### Superheated steam temperature

Figure 4b shows the measured steam temperature as a function of the solar flux. A maximum steam temperature of 144 °C was measured at 1.79 suns. At one sun, a steam temperature of 122 °C was achieved, marking the achievement of superheated steam generation at one sun illumination. Moreover, higher temperatures up to 133 °C were achieved by a simple radiative shielding method (“Shielded” results in Fig. 4), as detailed in the following section. Additionally, the CSES was able to generate superheated steam at temperatures above 111 °C at a solar flux of just 0.71 suns. The analytical model predicts the superheated steam temperature *T*_s_ according to

6 $$T_{\mathrm{s}} = T_{\mathrm{w}} + f_{{\mathrm{superheater}}}(T_{\mathrm{e}} - T_{\mathrm{w}})$$

where *f*_superheater_ is the superheater effectiveness (see Supplementary Notes 3 and 5), and *T*_e_ is given by Eq. (2). In the ideal case, the superheater effectiveness would be unity, such that the vapour is heated to the emitter temperature. In practice, the superheater is not a perfect heat exchanger (see Supplementary Note 5) such that there exists a finite temperature difference between the emitter and the exiting vapour. In our experiments, this temperature difference increased nearly linearly as a function of the solar flux, ranging between 5 °C at 0.7 suns and 9 °C at 1.5 suns. A value of *f*_superheater_ = 0.8 was found to give the best overall fit to the experimental data in Fig. 4b.

### Controlling the degree of superheating

In many applications, for example sterilization^30^, it is desirable to deliver the steam at a constant temperature. Figure 4b shows that the steam temperature depends on the incident solar flux *q̇*_solar_, with a functional form given by Eqs. (6) and (2). Delivering constant temperature steam is therefore challenging considering the intermittency and variability of solar radiation. In active solar thermal collectors, the outlet temperature can be controlled by changing the mass flow rate through the collector. In passive solar evaporators like the CSES, the mass flow rate is fixed by Eq. (3), and such control is not possible. However, Eqs. (6) and (2) also reveal that the steam temperature is a function of the gain heat transfer coefficient. Closer inspection of Eq. (2) reveals that the emitter temperature can be increased by decreasing the gain heat transfer coefficient. We therefore developed a simple method to control *U*_gain_, and thus the steam temperature, through radiative shielding (see Supplementary Note 12 for details). By placing a reflecting shield with a central hole (an aperture) between the emitter and water surface (see Supplementary Fig. 13), the view factor between the emitter and the water can be reduced, effectively decreasing the gain coefficient, and increasing the steam temperature. We demonstrated this in the laboratory using aluminium foil radiation shields with different central hole sizes (see Supplementary Fig. 6). The results of the “Shielded” experiments are highlighted in Fig. 4b, where a steam temperature of 133 °C was achieved at just 0.84 suns. This constitutes the highest temperature steam produced by a device operating at or below one sun illumination. Note that the increase in steam temperature is accompanied by a reduction of the system efficiency, as observed in Fig. 4a. This can be understood by examining the effect of reducing *U*_gain_ on the system efficiency c.f. Eq. (5).

We envision that active control of the steam temperature could be easily implemented through use of a simple iris diaphragm or Venetian blind type shutter acting as a variable radiation shield. The ability to easily control energy transfer is a unique feature of the contactless configuration that leverages the radiative mode of heat transfer. In a conventional solar evaporation structure, where heat is transferred to the water by thermal conduction, control of the gain heat transfer coefficient would entail changing the thermal conduction length, cross-sectional area, or thermal conductivity, a much more technically challenging proposition.

### Operation with sea water

**Fig. 5:**
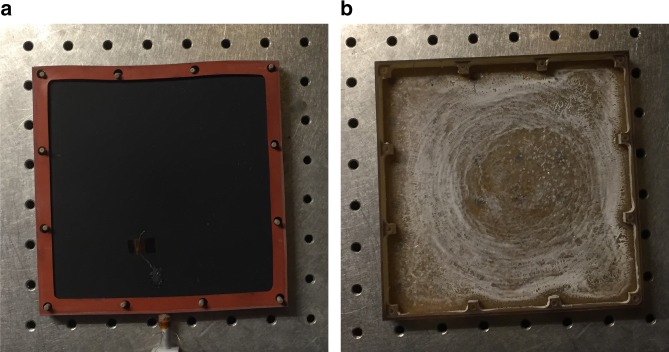
Operation with sea water. **a** Photograph of emitter after evaporation experiment with synthetic sea water (3.5 wt% NaCl dissolved in water) showing no evidence of fouling. **b** Photograph of the basin after evaporation experiment with synthetic sea water showing complete evaporation and containment of salt crystals to the basin

To demonstrate the fouling resistance of the CSES, we performed additional laboratory experiments using synthetic sea water (3.5 wt% NaCl in water). The sea water experiment was run at one sun for a period of 8 h, long enough to completely evaporate the entire 100 g of water in the reservoir. Figure 5a shows a photograph of the emitter surface after the experiment, showing no sign of salt fouling, e.g., salt crystal formation. Additionally, no fouling was observed in the RVC foam, absorber, or steam outlet, indicating that all salt was successfully contained in the basin. Figure 5b shows a photograph of the basin after the experiment, where crystals formed by salt precipitated out of the sea water are evident. No evidence of salt creeping upward along the basin sidewall above the height of the initial meniscus was observed. Any contamination or salt built-up on the basin following an experiment can be easily removed by flushing with water or even brine (e.g., sea water). Furthermore, any residual fouling of the basin is not expected to reduce the performance of the CSES since the water serves as its own absorber of radiant energy, with the basin serving no function other than to hold the water. Importantly, the fouling resistance results from the contactless design, rather than particular anti-fouling materials. Therefore, the contamination resistance is not specific to a particular salt, nor is it expected to degrade over time or in harsher environments. No significant changes in evaporation rate, steam temperature or efficiency were observed for the experiments performed with sea water. This is attributed to the fact that the mass flow rate is driven by Eq. (3), and that there are no significant differences in *h*_fg_ for pure water and brine assuming ideal solution behaviour^42^, at least until the point at which salt begins to precipitate. Owing to the high operating temperatures and the judicious choice of materials, the CSES additionally appears to be resilient to biofouling and corrosion induced fouling, and neither effects were observed during the 30 plus of experiments conducted with the device.

### Outdoor demonstration

**Fig. 6:**
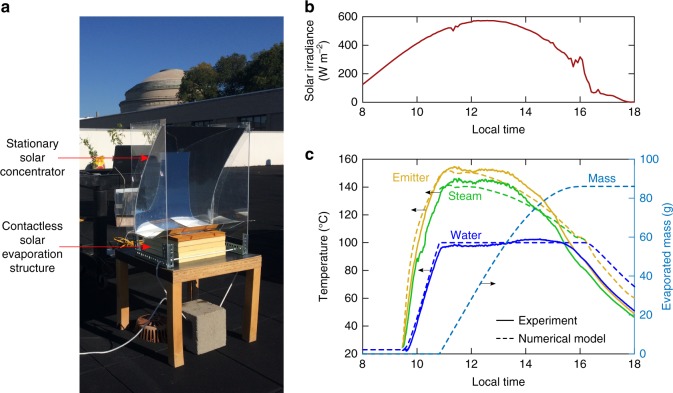
Outdoor operation of the contactless solar evaporation structure. **a** Photograph of the outdoor experiment on the MIT roof using a stationary (non-tracking) solar concentrator. **b** Solar irradiance (global horizontal) measured over the course of the test day, October 21, 2017. **c** Performance of the CSES during the outdoor experiment. Solid lines are measured values and dashed lines are modelled values from the transient model validated vis-à-vis the indoor laboratory experiments

Following the successful laboratory demonstrations, we performed outdoor experiments on the roof of MIT in Cambridge, Massachusetts, to demonstrate the ability of the CSES to generate superheated steam with natural sunlight. The main test day was October 21, 2017, during which the total solar flux reached a maximum value of 590 W/m^2^ (Fig. 6b). To augment the solar flux, we designed and constructed a low-cost stationary optical concentrator (Fig. 6a). The concentrator uses a truncated linear asymmetric compound parabolic concentrator (2D-ACPC) design^43^ (see Supplementary Note 13 and Supplementary Fig. 14) and achieves a geometric concentration ratio of 3.1, without the need for diurnal tracking. Only seasonal adjustment would be necessary for this optic, which significantly reduces the cost and complexity compared with higher concentration optical concentrators^6^. Figure 6a shows a photograph of the outdoor experimental setup, where the CSES sits at the exit of the low-cost stationary optical concentrator. Figure 6c shows the measured and modelled temperature and modelled mass evolution over the course of the outdoor experiment. The water reservoir reached a temperature of 100 °C in 1.5 h, despite the average solar flux being just 400 W m^−2^ during this heat-up phase. Following the heat-up phase, superheated steam was generated over a period lasting 3.5 h. Remarkably, steam temperatures in excess of 146 °C were recorded. The outdoor experiment, demonstrates the ability of the CSES device to generate superheated steam even during autumn days with low solar elevation and moderate solar fluxes.

Additional outdoor experiments were performed where the produced vapour was condensed in a flask and collected, allowing a secondary quantification of the amount of vapour produced. Collection experiments were performed in June and July 2018, without the use of a solar concentrator. The transient response of a representative run is given in Supplementary Fig. 15. During this experiment, 8.9 g of distilled water were collected, which agrees reasonably well with the measured mass loss of the water in the basin of to 12.8 g. The difference is partially accounted for by 1.6 g of residual water that were condensed on the emitter and in the outlet tubing and thus did not make it to the flask, with the remaining 2.3 g of vapour attributed to a small leak. A similar repeat experiment revealed 9.8 g collected water, 13.2 g evaporated from the basin and 1.6 g residual water. The maximum steam temperature recorded during the outdoor experiments with no solar concentrator was 117 °C. These experiments clearly show the ability of the CSES to produce superheated vapour, and easily collectable distilled water, under natural sunlight without the need for a solar concentrator.

### Evaporation mechanism

In previous solar evaporation structures which operate below the boiling point of water, evaporation is driven by a vapour concentration gradient between the water/vapour interface and the surroundings^44^. In contrast, the CSES brings the water reservoir is to its boiling point *T*_b_, opening up a new channel for evaporation (Supplementary Note 14). With the water at *T*_b_, additional energy added to the water will tend to raise its temperature, and, concomitantly, its saturation pressure. Importantly, the saturation pressure increases exponentially with water temperature implying that a small rise in temperature gives rise to a large vapour pressure surplus. When the flow resistance is small, the increase in vapour pressure easily drives the generated vapour through the superheater and outlet tube without a significant pressure buildup in the system. We measured the pressure drop through the CSES, and found it to be below 200 Pa for the flow rates encountered during typical operation (Supplementary Fig. 16). This corresponds to a boiling point elevation of just 0.06 K, indicating that the flow resistance of the RVC and CSES system is indeed very small.

Although the CSES operates at the boiling point, the evaporation mechanism is fundamentally different from boiling. In pool boiling^45^, the heat source is at a solid–water interface and excess temperature (wall superheat) is necessary nucleate a bubble which then rises to the cooler liquid–vapour interface. In the contactless configuration, the effective heat source is within less than 100 μm from the liquid–vapour interface, such that steam generation occurs by interfacial evaporation and bubble formation is not necessarily required. Given the unique characteristics elucidated here, we believe this work is of fundamental interest to the evaporation community.

### Design optimization

**Fig. 7:**
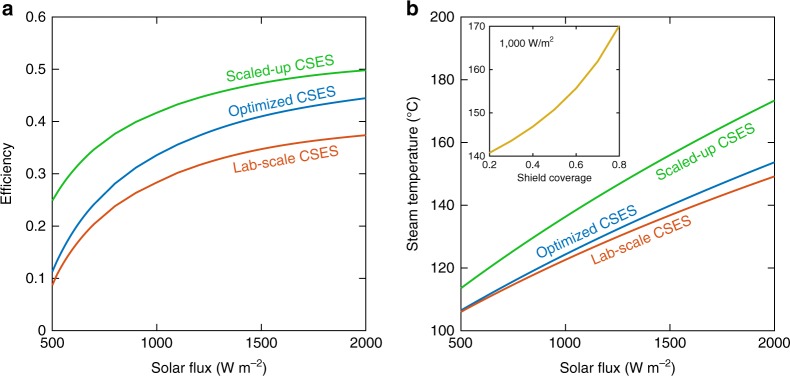
Performance of optimized and scaled-up contactless solar evaporation structure. **a** Steady-state efficiency as a function of the incident solar flux for as-tested lab-scale CSES, optimized CSES and scaled-up CSES as predicted by the numerical model. **b** Superheated steam temperature as a function of the incident solar flux for each design as predicted by the numerical model. The inset shows the effect of radiative shielding (see Supplementary Note 10 and Supplementary Fig. 12) on the steam temperature of the scaled-up design operating at one sun. The optimized and scaled-up designs utilizes a copper foam in lieu of the RVC foam to reduce the absorber/emitter temperature gradient. For the scaled-up design, it is assumed that the device can be made large enough such that side losses can be neglected and that the superheater effectiveness can be brought to unity

The lab-scale CSES exhibited a large temperature drop between the absorber and emitter (see Fig. 3). Insight gained from the numerical heat transfer model revealed that the main cause of this temperature drop is the low effective thermal conductivity of the RVC layer (0.05 W m^−2^K^−1^) due to the low bulk thermal conductivity of vitreous carbon^46^. This indicates that changing to a graphitic or metallic foam with higher effective thermal conductivity could significant increase the efficiency and superheated steam temperature. To test this hypothesis, we used the validated numerical model to estimate the performance of an “optimized CSES” with the RVC replaced by a copper foam having an effective thermal conductivity of 10.1 W m^−2^K^−1^. Figure 7 shows the efficiency and steam temperature for the optimized design in comparison with the as-tested lab-scale CSES. Also shown is the predicted performance for a “scaled-up CSES” which utilizes the copper foam, and where we have additionally assumed that the device is large enough such that side-losses are negligible and that superheater effectiveness is unity. For the optimized and scaled-up CSES designs operating at one sun, the model predicts efficiencies of over 33 and 41%, and steam temperatures of 124 °C and 136 °C, respectively. Moreover, higher steam temperatures could be achieved by the radiative shielding method proven in the lab-scale demonstrations, as shown in the inlay of Fig. 7b.

In summary, we demonstrated a method to generate steam using low intensity solar flux based on non-contact radiative transfer from the solar absorber to the water. In the contactless configuration, water serves as its own thermal absorber making the device extremely robust against contamination. This device could be used to generate clean distilled water from contaminated and high-salinity brines without risk of fouling or clogging. Operating at an efficiency of 25%, a CSES with a 1 m^2^ footprint could provide 2.5 litres of fresh water per day at a location with a daily insolation of 6 kWh/m^2^. Despite the higher heat losses associated with the higher temperatures achieved, the CSES offers promise as a solar desalination device due to the built-in collection which removes the need for a condensation cover. Additionally, the contactless configuration thermally decouples the absorber and the water, enabling the absorber to serve the dual function of a superheater, bringing the steam into the superheated state with temperatures in excess of 130 °C at one sun. Such temperatures make the device ideal for sterilization applications, where the CSES could be coupled to an autoclave to enable sterilization of medical equipment in remote locations with little access to electricity. Additionally, the achievable temperatures open up new applications including cooking, laundering, absorption/adsorption cooling and process heating. The demonstrated method of steam temperature control through radiative shielding would allow the device to output a constant steam temperature even during cloudy and low solar flux periods. Given the wide range of potential applications, we believe this demonstration of contactless solar steam generation will open up new avenues for harnessing solar energy and transforming it into useful forms.

## Methods

### Optical properties measurement

Optical properties of the relevant materials were measured over a broad wavelength range 250 nm to 25 μm by Ultraviolet-Visible-Near-infrared (UV-Vis-NIR) and Fourier Transform Infrared (FTIR) spectroscopy, using an Agilent Cary 5000 spectrophotometer and Thermo Fisher Nicolet 6700 Fourier transform infrared spectrometer, respectively. Details are presented in Supplementary Note 7.

### Laboratory experiments

Laboratory-scale experiments (detailed in Supplementary Note 15) were conducted by illuminating the device with simulated solar radiation from a Class AAA solar simulator (ScienceTech, SS-1.6 K, experimental setup shown in Supplementary Fig. 17). The average solar flux over the absorber was measured using a mapping technique (see Supplementary Fig. 18). In brief, the absolute solar flux at the centre of the absorber was measured using a thermopile detector (Newport, 818P-001-12) and power meter (Newport, 1918-C). A relative flux map over the absorber plane was measured by imaging a Lambertian target using a grayscale CMOS camera (Basler acA1920-25gm). The relative map was then scaled by equating the average pixel intensity over the thermopile detector area to the power meter reading, thus giving an absolute flux map (Supplementary Fig. 19). The total solar power input, and average solar flux, was then determined by integrating the absolute flux map over the absorber area. This procedure was repeated for every experiment to account for variation in solar simulator beam shape and device positioning.

A standard laboratory experiment consisted of filling the basin with 100 g of water, assembling the CSES and exposing it to simulated solar radiation for 5 to 18 h, depending on the solar flux, long enough to reach the steady-state region and measuring the temperature and mass evolution over the course of the experiment. Temperatures in the CSES were measured by precision fine-gauge K-type thermocouples (Omega 5TC-TT-K-36/40-36/72) connected to a data acquisition system (Omega DAQPRO 5300). The thermocouple locations are detailed in Supplementary Fig. 7. Due to difficulties in measuring gas temperatures in radiating environments, a custom-built radiation-shielded thermocouple suspended in the centre of the outlet tube was used to measure the superheated steam temperature. The estimated radiation error of the radiation-shielded thermocouple is below 1 K (see Supplementary Note 10). Evaporation rates were determined by placing the CSES device on a balance (A&D EJ3000) and measuring the mass loss over the course of the experiment. Steady-state efficiencies were based on the total mass loss determined by measuring the mass of water in the basin before and after the experiment, and correcting for mass loss during the heat-up phase based on the reading from the balance (see Supplementary Note 11).

### Outdoor experiments

Outdoor experiments were performed during autumn 2017 on the roof of Building 1 on MIT’s main campus in Cambridge, Massachusetts. A water mass of 150 g was used for the outdoor experiment. Solar flux (global horizontal irradiance) and outdoor temperature were measured using a HOBO U30 Weather Station, operated by the MIT Sustainable Design Lab. Temperatures were measured as in the laboratory experiments. Mass was not measured in the outdoor experiment due to wind disturbing the balance, however, the mass loss could be predicted using the validated transient model.

Outdoor experiments with collection were performed during summer 2018 at the same test location. During this time of year, the solar irradiance was high enough to permit operation of the CSES without the need for a solar concentrator. A long FEP tube was connected to the vapour outlet and directed into a volumetric flask. The flask was placed into an ice bath to promote condensation of the superheated vapour onto the flask wall. To reduce the chance of evaporation of the condensed vapour into the environment, the top of the flask was sealed with tape except for a small puncture hole to avoid pressure buildup. The amount of condensed vapour collected in the flask was measured by weighing the volumetric flask before and after emptying it. A water mass of 50 g was used to reduce the heat up time relative to the lower solar input. For the collection experiments, solar flux was measured using a Hukseflux LP-02 pyranometer.

### Modeling

The transient coupled heat and mass transfer model was formulated using a quasi-one-dimensional equivalent circuit technique, and implemented in the Simulink^®^ Simscape^TM^ environment. Details of the model are described in Supplementary Note 5. Mass transfer was implemented according to Eq. (3) and coupled to the heat transfer circuit through a custom-built block in Simscape^TM^. Multidimensional effects were accounted for through a distributed resistance arrangement, informed by a steady-state multidimensional finite element heat transfer model implemented in Solidworks Simulation (see Supplementary Note 16 and Supplementary Figs. 20 and 21). In brief, the transient model breaks the system down into a set of discrete thermal capacitances and thermal resistances, which may be either linear in temperature (thermal convection and thermal conduction resistances) or nonlinear in temperature (radiation resistances). The boundary conditions are the absorbed solar power input and the environment temperature, and the initial conditions are the initial water mass and initial temperature of the system. The resulting set of ordinary differential equations is solved using the Runge–Kutta technique with trapezoidal integration, as implemented by the ode23t solver in Simulink^®^. The simulation time for a single run is ~0.5 s.

## Electronic supplementary material

Supplementary Information

Description of Additional Supplementary Files

Supplementary Movie 1

## Data Availability

All relevant data will be made available upon reasonable request. Requests for data should be addressed to G.C.
