# Supplementary material for: Contactless steam generation and superheating under one sun illumination
Source: Nat Commun. 2018 Dec 11;9:5086. doi: 10.1038/s41467-018-07494-2 (PMC6290071; doi:10.1038/s41467-018-07494-2)
Supplement: Supplementary file 1 — Supplementary Information [file 41467_2018_7494_MOESM1_ESM.pdf]

Supplementary Information

for

Contactless steam generation and superheating under one sun illumination

Thomas A. Cooper, Seyed H. Zandavi, George W. Ni, Yoichiro Tsurimaki, Yi Huang, Svetlana V. Boriskina, Gang Chen*

Department of Mechanical Engineering

Massachusetts Institute of Technology

77 Massachusetts Ave., Cambridge, Massachusetts 02139, USA

*e-mail: gchen2@mit.edu

Contents

Supplementary Note 1. Nomenclature **4**
Supplementary Note 2. Water optical and radiative properties **10**
Internal absorption 10
Emittance 11
Supplementary Note 3. Steady-state analytical model **13**
Energy balance and efficiency 13
Superheating 15
Summary of parameters in steady-state model 17
Supplementary Note 4. Optical efficiency **18**
Supplementary Note 5. Transient numerical model **19**
Nominal mass flow rate 21
Emitter-water radiative exchange 21
Gas gap convection 22
Sidewall conduction 22
Biot number and water temperature distribution 22
Superheater heat transfer 23
Multilayer polymer glazing 25
Radiation losses 26
Top, bottom, and side losses 26
Other losses 27
Supplementary Note 6. Details of the CSES design **28**
CSES device 28
Multilayer polymer glazing 29
Experimental assembly 29
Supplementary Note 7. Optical properties measurement **31**
Selective surface 31
Advanced FEP glazing material 31
Emitter 32
Supplementary Note 8. Reducing transient heat-up time **33**
Supplementary Note 9. Convection in the gas gap **34**
Supplementary Note 10. Radiation-shielded thermocouple **36**
Supplementary Note 11. Details of efficiency calculation **38**
Supplementary Note 12. Controlling the degree of superheat **39**

Supplementary Note 13. Details of the outdoor experiments with stationary concentrator 40
Supplementary Note 14. Evaporation mechanism 41
Mass diffusion resistance 41
Flow resistance 42
Interface resistance 43
Supplementary Note 15. Details of the laboratory experiments 45
Supplementary Note 16. Multidimensional conduction model 48
Supplementary Figures 50
Supplementary Tables 71
Supplementary References 72

Supplementary Note 1. Nomenclature

Latin characters

A	Footprint area (15.24 cm × 15.25 cm)
$A_{\text{cond,RVC}}$	Thermal conduction area of RVC (m^2)
A_{e}	Emitter area (m^2)
A_{h}	Radiation shield hole area (m^2)
AAF	Active area fraction
a_{i}	Concentrator inlet aperture width (m)
a_{o}	Concentrator outlet aperture width (m)
A_{sidewall}	Sidewall conduction area (m^2)
$A_{\text{solid-vapour,RVC}}$	RVC solid-vapour heat exchange area (m^2)
A_{TC}	Surface area of thermocouple tip (m^2)
A_{tube}	Inner cross-sectional area of outlet tube (m^2)
A_{w}	Area of water/vapour interface (m^2)
$A_{\text{wire cross-section}}$	Cross-sectional area of thermocouple wire (m^2)
Bi	Biot number
C	Thermal capacitance (J/K)
C_1	Constant of integration
C_2	Constant of integration
C_{g}	Geometric concentration ratio
$\dot{C}_{\text{max}}$	Maximum heat capacitance rate (W/K)
$\dot{C}_{\text{min}}$	Minimum heat capacitance rate (W/K)
c_{p}	Constant pressure specific heat (J kg^{-1} K^{-1})
$c_{\text{p,basin}}$	Constant pressure specific heat of basin (J kg^{-1} K^{-1})
$c_{\text{p,s}}$	Constant pressure specific heat of steam (J kg^{-1} K^{-1})
$c_{\text{p,w}}$	Constant pressure specific heat of water (J kg^{-1} K^{-1})
C_{shield}	Shield coverage ratio
$C_{\text{v},\infty}$	Ambient molar concentration of water vapour (mol/m^3)
$C_{\text{v,inside}}$	Molar concentration of water vapour inside the device (mol/m^3)
dHI	Diffuse horizontal irradiance (W/m^2)
DNI	Direct normal irradiance (W/m^2)
d_{pore}	RVC pore diameter (m)
d_{TC}	Diameter of thermocouple tip (m)
D_{TC}	Inner diameter of thermocouple shield (m)
d_{w}	Depth of water reservoir in basin (m)
$D_{\text{v-air}}$	Binary diffusion coefficient of water vapour and air (m^2/s)
$f_{\text{superheater}}$	Superheater effectiveness
$F_{\text{e-sidewall}}$	Radiation view factor from emitter to basin sidewall
$F_{\text{e-w}}$	Radiation view factor from emitter to water
$F_{\text{w-sidewall}}$	Radiation view factor from water to basin sidewall
g	Gravitational acceleration (m/s^2)
G	Radiation coefficient (W/K^4)
Gr	Grashoff number

GV	Grayscale value
GV(x,y)	Grayscale value at pixel coordinate (x,y)
$\langle GV \rangle_{ROI_1}$	Average grayscale value over thermopile area
$\langle GV \rangle_{ROI_2}$	Average grayscale value over absorber area
h_f	Specific enthalpy of water (J/kg)
h_{fg}	Latent heat of vaporization (J/kg)
h_g	Specific enthalpy of steam (J/kg)
$h_{sensible}$	Specific sensible heat of steam (J/kg)
i	Imaginary unit
I	Intensity (W m^{-2}sr^{-1})
I_0	Intensity at L = 0 (W m^{-2}sr^{-1})
I_λ	Spectral intensity (W m^{-2}sr^{-1}μm^{-1})
$I_{\lambda,0}$	Spectral intensity at L = 0 (W m^{-2}sr^{-1}μm^{-1})
J	Radiosity (W m^{-2})
$\bar{J}_{coll}$	Collision frequency (mol s^{-1}m^{-2})
J_e	Emitter radiosity (W m^{-2})
J_e	Sidewall radiosity (W m^{-2})
j_s	Steam mass flux (kg s^{-1}m^{-2})
J_w	Water radiosity (W m^{-2})
k	Imaginary part of the refractive index
k	Thermal conductivity (W m^{-2}K^{-1})
k_{air}	Thermal conductivity of air (≈0.03 W m^{-2}K^{-1})
$k_{eff,RVC}$	Effective thermal conductivity of RVC (0.05 W m^{-2}K^{-1})
k_{PEI}	Basin thermal conductivity (0.22 W m^{-2}K^{-1})
k_s	Thermal conductivity of steam (≈0.025 W m^{-2}K^{-1})
k_{TC}	Thermal conductivity of thermocouple wire (30 W m^{-2}K^{-1})
k_w	Thermal conductivity of water (≈0.67 W m^{-2}K^{-1})
L	Depth in Beer-Lambert law (m)
L_{90}	Depth for 90% internal absorption (m)
$L_{cond,RVC}$	Thermal conduction length in RVC (m)
$L_{gas\ gap}$	Gas gap size (m)
$L_{glazing\ gap}$	Glazing layer separation (m)
$L_{sidewall}$	Sidewall conduction length (m)
L_{TC}	Protrusion length of thermocouple tip (m)
L_{tube}	Length of the outlet tube (m)
L_{top}	Length used for calculation of top heat losses (m)
m	Mass (kg)
$\dot{m}$	Steam mass flow rate (evaporation rate) (g/s)
m_{basin}	Mass of basin (m)
m_w	Mass of water (m)
M_w	Molar mass of water (kg/mol)
$\tilde{n}$	Complex refractive index
N	Number of layers in glazing system

n	Real part of the refractive index
NTU	Number of transfer units
Nu	Nusselt number
p	Total pressure (Pa)
p_{inside}	Total pressure inside the CSES device (Pa)
p_{v}	Water vapour partial pressure just above the interface (Pa)
p_{∞}	Ambient total pressure (Pa)
Pe	Péclet number
Pr	Prandtl number
p_{sat}	Saturation pressure (Pa)
$\dot{Q}$	Heat transfer rate (W)
Q	Thermal energy (J)
$\dot{Q}_{\text{1D}}$	Heat losses assuming one-dimensional heat transfer (W)
$\dot{Q}_{\text{3D}}$	Heat losses from three-dimensional model (W)
$\dot{q}_{\text{abs}}$	Absorbed solar heat flux (W/m^2)
$\dot{Q}_{\text{abs}}$	Absorbed solar power input (W)
$\dot{q}_{\text{bb},\lambda}$	Blackbody spectral hemispherical emissive power (W/m^2)
$\dot{q}_{\text{cond}}$	Conduction heat flux (W/m^2)
$\dot{q}_{\text{conv}}$	Convection heat flux (W/m^2)
$\dot{q}_{\text{gain}}$	Net heat flux to water (W/m^2)
$\dot{q}_{\text{loss}}$	Heat flux lost to environment (W/m^2)
$\dot{Q}_{\text{max}}$	Maximum heat transfer rate (W)
$\dot{q}_{\text{o}}$	Average flux at the outlet of the concentrator (W/m^2)
$\dot{q}_{\text{rad}}$	Radiation heat flux (W/m^2)
$\dot{Q}_{\text{rad}}$	Radiation heat transfer rate (W)
$\dot{q}_{\text{s}}$	Heat flux transferred to steam (W/m^2)
$\dot{q}_{\text{s,max}}$	Maximum heat flux transferred to steam (W/m^2)
$\dot{q}_{\text{solar}}$	Incident solar heat flux (W/m^2)
$\dot{q}_{\text{solar}}(x,y)$	Solar heat flux at pixel coordinate (x,y) (W/m^2)
$\dot{q}_{\text{solar,0}}$	Break-even incident solar heat flux (W/m^2)
$\dot{q}_{\text{solar},\lambda}$	Spectral solar irradiance (W m^{-2}μm^{-1})
$\dot{q}_{\text{thermopile}}$	Heat flux measured by thermopile (W m^{-2})
$\dot{Q}_{\text{top}}$	Top heat losses (W)
$\dot{Q}_{\text{tot}}$	Total heat losses (W)
$\dot{q}_{\text{w}}$	Conduction/convection heat flux to water (W/m^2)
R	Thermal resistance (K/W)
Ra	Rayleigh number
R_{cond}	Conduction resistance for shielded thermocouple (K/W)
$R_{\text{cond,RVC}}$	Thermal conduction resistance of RVC (K/W)
Re	Reynolds number
R_{tot}	Total thermal resistance (K/W)
$R_{\text{rad,tot}}$	Total radiation resistance (m^{-2})
R_{u}	Ideal gas constant (J mol^{-1}K^{-1})
sf	Scaling factor for flux map

SSA_{RVC}	Specific surface area of RVC (m^2/m^3)
T	Temperature (K)
t	Time (s)
T_∞	Ambient temperature (K)
T_a	Absorber temperature (K)
T_A	Nodal temperature (K)
T_b	Boiling point (K)
T_B	Nodal temperature (K)
T_{bb}	Blackbody temperature (K)
$T_{C,in}$	Inlet temperature of colder fluid (K)
T_e	Emitter temperature (K)
$T_{H,in}$	Inlet temperature of hotter fluid (K)
T_m	Intermediate temperature in radiation heat transfer coefficient (K)
T_{ref}	Reference temperature (K)
T_s	Steam temperature (K)
T_{sat}	Saturation temperature (K)
$T_{sidewall}$	Basin sidewall temperature (K)
T_{TC}	Thermocouple temperature (K)
T_v	Vapour temperature just above the water/vapour interface (K)
T_w	Water temperature (K)
u	Steam velocity (m/s)
UA_{RVC}	Superheater overall heat transfer coefficient area product (W/K)
U_{bot}	Overall heat transfer coefficient for bottom losses (W m^{-2} K^{-1})
$U_{cond,TC}$	Shielded thermocouple conduction effective heat transfer coefficient (W m^{-2} K^{-1})
U_{conv}	Convection heat transfer coefficient (W m^{-2} K^{-1})
$U_{conv,gas\ gap}$	Convection heat transfer coefficient in the gas gap (W m^{-2} K^{-1})
$U_{conv,glazing}$	Convection heat transfer coefficient for the glazing system (W m^{-2} K^{-1})
$U_{conv,TC}$	Shielded thermocouple convection heat transfer coefficient (W m^{-2} K^{-1})
U_{gain}	Gain overall heat transfer coefficient (W m^{-2} K^{-1})
$U_{gain,rad}$	Gain radiation heat transfer coefficient (W m^{-2} K^{-1})
U_{loss}	Loss overall heat transfer coefficient (W m^{-2} K^{-1})
$U_{rad,TC}$	Shielded thermocouple radiation heat transfer coefficient (W m^{-2} K^{-1})
U_{side}	Overall heat transfer coefficient for side losses (W m^{-2} K^{-1})
$U_{solid\text{-}vapour,RVC}$	RVC solid-vapour heat transfer coefficient (W m^{-2} K^{-1})
U_{top}	Overall heat transfer coefficient for top losses (W m^{-2} K^{-1})
$\dot{V}$	Volumetric flow rate (m^3/s)
V_{RVC}	Volume of RVC (m^3)
w_{basin}	Basin width (m)
X	Ratio of side length to separation of directly opposed squares
x,y	Pixel coordinates (m)
z	Vertical coordinate (m)

Greek characters

α	Absorptance

α_s	Thermal diffusivity of steam
α_{solar}	Solar weighted absorptance
α'_λ	Spectral directional absorptance
β	Volumetric thermal expansion coefficient (K^{-1})
β_λ	Spectral extinction coefficient (m^{-1})
$\Delta m_{heat\text{-}up}$	Mass loss during the heat-up phase (g)
$\Delta m_{basin,heat\text{-}up}$	Mass loss during the heat-up phase due to evaporation of water in the basin (g)
Δm_{basin}	Difference in mass of water in the basin before and after experiment (g)
Δm_{ss}	Evaporated mass during the quasi-steady-state region (g)
ΔT	Temperature difference (K)
ΔT_{bot}	Water to ambient temperature difference (K)
ΔT_{top}	Absorber to ambient temperature difference (K)
Δt_{ss}	Duration of the quais-steady-state region
ϵ'	Spectral hemispherical emittance
ϵ	Total hemispherical emittance
ϵ_e	Emittance of emitter
ϵ_{eff}	Effective emittance
$\epsilon_{eff,top}$	Effective emittance of selective surface
$\epsilon_{eff,w}$	Effective emittance of water with shield
ϵ_{sh}	Emittance of radiation shield
ϵ_{ss}	Emittance of selective surface
$\epsilon_{sidewall}$	Emittance of basin sidewall
ϵ_{TC}	Emittance of thermocouple tip
ϵ_w	Emittance of water
ϵ'_λ	Spectral directional emittance
η	Efficiency
η_{max}	Maximum efficiency
η_{opt}	Optical efficiency
η_{th}	Thermal efficiency
θ	Angle of incidence (deg)
ϑ	Solar incidence angle (deg)
θ_i	Concentrator acceptance angle (deg)
κ_λ	Spectral absorption coefficient (m^{-1})
λ	Wavelength (μm)
μ_s	Dynamic viscosity of steam (Pa s)
ν	Kinematic viscosity (m^2/s)
ρ	Density (kg/m^3)
ρ_{mirror}	Reflectance of concentrator mirror
ρ_s	Density of steam (kg/m^3)
ρ'_λ	Spectral directional-hemispherical reflectance
$\rho'_{\lambda,para}$	ρ'_λ for parallel polarization
$\rho'_{\lambda,perp}$	ρ'_λ for perpendicular polarization
σ	Stefan-Boltzmann constant (5.67×10^{-8} W $m^{-2}K^{-4}$)
σ_e	Evaporation coefficient in Hertz-Knudsen equation

σ_c	Condensation coefficient in Hertz-Knudsen equation
τ	Transmittance
τ'_{int}	Directional internal transmittance
τ_{IR}	Infrared transmittance
τ_N	Transmittance of an *N*-layer glazing system
τ_{solar}	Solar weighted transmittance
φ	Azimuthal angle (deg)
ϕ	Relative humidity
ω	Solid angle (sr)

Supplementary Note 2. Water optical and radiative properties

In this note, the relevant optical properties for liquid water including penetration depth, internal transmittance, and emittance are defined and calculated.

Internal absorption

The attenuation of a narrow monochromatic beam of radiation as it propagates through a medium is given by the Beer-Lambert law

$$I_{\lambda}(L) = I_{\lambda,0} e^{-\beta_{\lambda} L} \tag{1}$$

where $I_{\lambda}(L)$ is the spectral intensity a distance L into the medium, $I_{\lambda,0}$ is the spectral intensity at $L = 0$, and β_{λ} is the spectral extinction coefficient. In the absence of scattering, the extinction coefficient is equal to the absorption coefficient κ_{λ}, which can be found from the imaginary part k of the complex refractive index $\tilde{n} = n - ik$

$$\kappa_{\lambda} = \frac{4\pi k(\lambda)}{\lambda} \tag{2}$$

The reciprocal of the absorption coefficient $1/\kappa_{\lambda}$ can be interpreted as the absorption mean free path (MFP) for a photon of wavelength λ. When considering broadband radiation, e.g. solar or blackbody radiation, it is necessary to spectrally average the absorption coefficient. There are numerous ways to represent the spectrally averaged absorption coefficient or pathlength, the most common being the Planck-mean and Rosseland-mean absorption coefficients. For quantifying the absorbing capability of a material, it is most useful to consider the transmittance at a given depth. For a narrow beam of radiation, the internal transmittance at a given depth into the medium is found from

$$\tau'_{\text{int}}(L) = \frac{I(L)}{I_0} = \frac{\int_0^{\infty} I_{\lambda}(L) d\lambda}{\int_0^{\infty} I_{\lambda,0} d\lambda} = \frac{\int_0^{\infty} I_{\lambda,0} e^{-\kappa_{\lambda} L}}{I_0} \tag{3}$$

Alternatively, Supplementary Eq. (3) can be solved for the depth L at which a certain fraction of the incident beam has been absorbed. For example, the 90% absorption depth L_{90} is the solution of Supplementary Eq. (3) for which the transmittance equals $1 - 0.9 = 0.1$. Supplementary Eq. (3) is for a narrow beam parallel to L. Supplementary Fig. 1 shows the resulting 90% absorption depth

for water for normally incident solar radiation and blackbody radiation at different emitter temperatures. For a diffuse beam

$$\tau_{\text{int}}(L) = \frac{\int_0^{\infty} \int_{\text{hemi}} I_{0,\lambda} \cos\theta \exp(-\kappa_\lambda L \sec\theta) \, d\omega d\lambda}{\pi I_0} \tag{4}$$

where $d\omega$ is a differential element of solid angle and hemi denotes integration over the hemisphere. Due to the diffuse nature of thermal radiation vs. the collimated nature of solar radiation, the required absorption depths for a thermal source tend to be even lower than those given in Supplementary Fig. 1.

Emittance

Water is a good internal absorber of thermal radiation. However, the absorptance can never reach 100% due to Fresnel reflection at the air/water interface. Assuming the water depth is large enough to make the transmittance sufficiently close to zero at the wavelength of interest, the spectral directional emittance ϵ'_λ of an optically thick (i.e. $\tau'_{\text{int}} \to 0$) layer of water may be determined from

$$\epsilon'_\lambda = \alpha'_\lambda = 1 - \rho'_\lambda = 1 - \frac{1}{2}\left(\rho'_{\lambda,\text{para}} + \rho'_{\lambda,\text{perp}}\right) \tag{5}$$

where α'_λ is the spectral directional absorptance, ρ'_λ is the spectral directional-hemispherical reflectance, and the para and perp subscripts indicate parallel and perpendicular polarizations respectively. The first equality in Supplementary Eq. (5) follows from Kirchhoff's law, the second equality results from an energy balance considering $\tau'_\lambda \to 0$, and the final equality results from the assumption of equal proportions of parallel and perpendicular polarizations for a thermal source. The polarized spectral directional-hemispherical reflectance can be found from the Fresnel reflectance formulae

$$\rho'_{\lambda,\text{para}} = \left| \frac{\tilde{n}^2 \cos\theta_{\text{i}} - \sqrt{\tilde{n}^2 - \sin^2\theta}}{\tilde{n}^2 \cos\theta_{\text{i}} - \sqrt{\tilde{n}^2 - \sin^2\theta}} \right|^2 \tag{6}$$

$$\rho'_{\lambda,\text{perp}} = \left| \frac{-\cos\theta_{\text{i}} + \sqrt{\tilde{n}^2 - \sin^2\theta}}{\cos\theta_{\text{i}} + \sqrt{\tilde{n}^2 - \sin^2\theta}} \right|^2 \tag{7}$$

where $\tilde{n}$ is the wavelength-dependent complex refractive index and θ is the incidence angle. The spectral hemispherical emittance can be determined by directionally averaging Supplementary Eq. (5) assuming diffuse (Lambertian) directional distribution

$$\epsilon_\lambda = \frac{\int_{\varphi=0}^{2\pi}\int_{\theta_i=0}^{\pi/2} \varepsilon'_\lambda(\lambda,\theta)\cos\theta\sin\theta d\theta d\varphi}{\int_{\varphi=0}^{2\pi}\int_{\theta_i=0}^{\pi/2} \cos\theta\sin\theta d\theta d\varphi} = 2\int_{\theta_i=0}^{\pi/2} \epsilon'_\lambda(\lambda,\theta)\cos\theta\sin\theta d\theta \quad (8)$$

The resulting spectral hemispherical emittance of water is given in Supplementary Fig. 2a. Also shown is the spectral hemispherical emissive power for a blackbody at 100 °C.

The total hemispherical emittance can be calculated by spectrally weighting by the blackbody spectrum

$$\epsilon(T_{\mathrm{bb}}) = \frac{1}{\sigma T_{\mathrm{bb}}^4}\int_0^\infty \epsilon_\lambda \dot{q}_{\mathrm{bb},\lambda}(T_{\mathrm{bb}})d\lambda \quad (9)$$

where $\dot{q}_{\mathrm{bb},\lambda}$ is the spectral hemispherical emissive power for a blackbody at temperature T_{bb}, which follows the Planck distribution. Supplementary Fig. 2b shows the resulting total hemispherical emittance for water in the temperature range 0 to 100 °C.

Supplementary Note 3. Steady-state analytical model

In this note, a simple steady-state analytical model for the Contactless Solar Evaporation Structure (CSES) is derived. This model illustrates the operating principle of the CSES and the effect of the most important parameters on performance. Supplementary Eqs. (17) and (25) along with the parameters in Supplementary Table 1 give the main results of the model which is capable of predicting the efficiency and steam temperature.

Energy balance and efficiency

Consider a generic device, shown schematically in Supplementary Fig. 3a, comprising a solar absorber that transfers heat to a body of water causing it to evaporate. Assume that the device is perfectly insulated on the bottom and sides and that the length and width are large compared to the thickness such that all heat transfer processes are one-dimensional in the vertical direction. A per-unit-area energy balance on the absorber/emitter yields

$$\dot{q}_{\text{abs}} = \eta_{\text{opt}} \dot{q}_{\text{solar}} = \dot{q}_{\text{loss}} + \dot{q}_{\text{gain}} + \dot{q}_{\text{superheat}} \tag{10}$$

where $\dot{q}_{\text{abs}}$ is the absorbed solar flux, η_{opt} is the optical efficiency, $\dot{q}_{\text{solar}}$ is the incident solar flux, q_{loss} is the rate of heat loss to the environment per unit absorber area, $\dot{q}_{\text{gain}}$ is the rate of heat transferred to the water per unit absorber area, and $\dot{q}_{\text{superheat}}$ is the rate of heat per unit absorber area associated with superheating of the generated steam. For the moment we will neglect $\dot{q}_{\text{superheat}}$, as it is small compared to the other terms (see discussion later in this note). Defining the effective overall heat transfer coefficient, $U \equiv \dot{q}/\Delta T$, Supplementary Eq. (10) becomes (using the shorthand e = emitter, w = water, and later s = steam)

$$\eta_{\text{opt}} \dot{q}_{\text{solar}} = U_{\text{loss}} \left(T_{\text{e}} - T_{\infty}\right) + U_{\text{gain}} \left(T_{\text{e}} - T_{\text{w}}\right) \tag{11}$$

In this simple analytical model, it assumed that U is a constant such that Supplementary Eq. (11) remains linear. Nonlinear effects are considered in the transient numerical model described in Supplementary Note 5. For radiation, this requires linearizing the heat transfer coefficient according to

$$U_{\text{gain,rad}} = \frac{\sigma}{1/\epsilon_{\text{e}} + 1/\epsilon_{\text{w}} - 1} \left(T_{\text{e}}^2 + T_{\text{w}}^2\right)\left(T_{\text{e}} + T_{\text{w}}\right) = \frac{\sigma}{1/\epsilon_{\text{e}} + 1/\epsilon_{\text{s}} - 1} 4T_{\text{m}}^3 \tag{12}$$

where ϵ_e and ϵ_w are the emitter and water emittances respectively, and T_e and T_w are representative emitter and water temperature, or alternatively T_m is a representative intermediate temperature.

Assuming η_{opt}, $\dot{q}_{solar}$, U_{loss}, U_{gain} and T_∞ are known, Supplementary Eq. (11) contains two unknowns T_e and T_w in one equation. However, if the heat flux is sufficiently high (higher than the break-even flux defined later), then the water will reach its boiling point, $T_w = T_b = 100$ °C. At this point the water temperature will be pinned at T_b, and any additional energy delivered to the water will go towards phase change (evaporation) rather than further raising the temperature of the water. The minimum input flux for this to occur is that for which $\dot{q}_{gain} = 0$, i.e. the absorbed flux just balances the losses. At this break-even point, the heat transfer to the water is zero, which implies $T_e = T_w$. The break-even flux can be found from

$$\dot{q}_{solar,0} = U_{loss}\left(T_w - T_\infty\right)/\eta_{opt} \tag{13}$$

At any flux level $\dot{q}_{solar} > \dot{q}_{solar,0}$, the water will be pinned at is boiling point and any additional heat absorbed by the water will go towards evaporation at the liquid/vapour interface. Therefore, the evaporation process is heat transfer limited, and the evaporation is a function of the heat flux to the water alone

$$j = \frac{\dot{m}}{A} = \frac{\dot{q}_{gain}}{h_{fg}} \tag{14}$$

where j is the steam mass flux (mass flow rate $\dot{m}$ per unit absorber area A) and h_{fg} is the latent heat of vaporization. We are only interested in operating the device at flux levels $\dot{q}_{solar} > \dot{q}_{solar,0}$, such that the evaporation rate is non-negligible, and will therefore assume $T_w = T_b = 100$ °C for the remainder of the analysis. With T_w known, Supplementary Eq. (11) can be solved for the emitter temperature

$$T_e = \frac{\eta_{opt}\dot{q}_{solar} + U_{loss}T_\infty + U_{gain}T_w}{U_{loss} + U_{gain}} \tag{15}$$

The efficiency of the device (ignoring steam superheat to allow comparison with previous work) is defined as[1]

$$\eta = \frac{j \cdot h_{fg}}{\dot{q}_{solar}} = \frac{\dot{q}_{gain}}{\dot{q}_{solar}} = 1 - \frac{\dot{q}_{loss}}{\dot{q}_{solar}} \tag{16}$$

Substituting in Supplementary Eq. (15), we can solve for the efficiency as a function of $\dot{q}_{solar}$

$$\eta = \frac{U_{\text{gain}}\left(T_{\text{e}} - T_{\text{w}}\right)}{\dot{q}_{\text{solar}}} = \frac{U_{\text{gain}}}{U_{\text{loss}} + U_{\text{gain}}}\left(\eta_{\text{opt}} - U_{\text{loss}}\frac{T_{\text{w}} - T_{\infty}}{\dot{q}_{\text{solar}}}\right) \tag{17}$$

This very general expression for thermal efficiency holds for any passive solar evaporator driven by an input flux $\dot{q}_{\text{solar}}$ which must deliver heat at T_{w}. The generic efficiency curve is plotted in Supplementary Fig. 3b and is bound by two characteristic points: the maximum thermal efficiency

$$\eta_{\text{max}} = \frac{U_{\text{gain}}}{U_{\text{loss}} + U_{\text{gain}}}\eta_{\text{opt}} \tag{18}$$

and the break-even flux given by Supplementary Eq. (13). The efficiency curve can be written in terms of the maximum efficiency and the break-even flux

$$\eta = \eta_{\text{max}}\left(1 - \frac{\dot{q}_{\text{solar,0}}}{\dot{q}_{\text{solar}}}\right) \tag{19}$$

The efficiency can also be broken down into the optical and thermal efficiencies

$$\eta = \eta_{\text{opt}}\eta_{\text{th}} = \eta_{\text{opt}}\underbrace{\frac{U_{\text{gain}}}{U_{\text{loss}} + U_{\text{gain}}}\left(1 - \frac{U_{\text{loss}}\left(T_{\text{w}} - T_{\infty}\right)}{\dot{q}_{\text{abs}}}\right)}_{\eta_{\text{th}}} \tag{20}$$

Importantly, this analysis shows that the thermal efficiency cannot be increased to unity by increasing the solar flux, since as $\dot{q}_{\text{solar}} \to \infty$, $\eta_{\text{th}} \to U_{\text{gain}}/(U_{\text{gain}} + U_{\text{loss}})$. This is a characteristic of the passive nature of the device, resulting from the fact that as $\dot{q}_{\text{solar}}$ is increased, the absorber temperature, c.f. Supplementary Eq. (15), and thus the losses, are increased. This is in contrast to an active system, where the temperature can be controlled by increasing the mass flow rate. However, we will later show that in the contactless configuration, it is possible to control the emitter temperature through radiative shielding.

Superheating

The steady-state model presented thus far can predict the steady-state emitter temperature and evaporation efficiency. In the present analysis, the achievable superheat of the steam is also of interest. From the second law of thermodynamics, the absorber must be the hottest part of the system, and therefore the steam can, in theory, be maximally heated to the absorber (emitter) temperature.

Steam superheating is achieved by forcing the generated steam to pass through the absorber, enabling solid-vapour heat transfer which heats the steam beyond its saturation temperature. To be strict, the sensible heat transferred to the steam would need to be included in the energy balance in Supplementary Eq. (10). However, it can be shown that for moderate superheats, the sensible heat is relatively small compared to the latent heat of vaporization. As a representative case, consider a case where the steam is superheated to T_s = 160 °C. The sensible heat is[2]

$$h_{\text{sensible}} = h_{\text{g}}\left(160\ ^\circ\text{C}\right) - h_{\text{g}}\left(100\ ^\circ\text{C}\right) = 2796.2\ \text{kJ/kg} - 2676.2\ \text{kJ/kg} = 120.0\ \text{kJ/kg} \tag{21}$$

where h_g is the specific enthalpy of the vapour at a given temperature which can be found from standard steam tables. The latent heat is[2]

$$h_{\text{fg}}\left(100\ ^\circ\text{C}\right) = 2257.0\ \text{kJ/kg} \tag{22}$$

We find that the sensible heat amounts to just over 5% of the magnitude of the latent heat. Therefore, it is reasonable to use Supplementary Eq. (15) to calculate the emitter temperature even for the case when the emitter is used to superheat the steam. Supplementary Eq. (15) therefore gives the maximum possible superheated steam temperature for the device. The maximum amount of heat that can be transferred to the steam is therefore

$$\dot{q}_{\text{s,max}} = j \cdot c_{\text{p,s}}\left(T_{\text{e}} - T_{\text{w}}\right) \tag{23}$$

In reality, less heat will be transferred to the steam and the steam will not completely reach T_e. Following heat exchanger analysis methods[3], we define the superheater effectiveness $f_{\text{superheater}}$ as the ratio of the actual amount of heat transferred to the steam to the maximum given by Supplementary Eq. (23) such that

$$\dot{q}_{\text{s}} = f_{\text{superheater}} \cdot j \cdot c_{\text{p,s}}\left(T_{\text{e}} - T_{\text{w}}\right) \tag{24}$$

The resulting superheated steam temperature is

$$\begin{aligned} T_{\text{s}} &= T_{\text{w}} + \frac{\dot{q}_{\text{s}}}{j \cdot c_{\text{p,s}}}\left(T_{\text{e}} - T_{\text{w}}\right) = T_{\text{w}} + f_{\text{superheater}}\left(T_{\text{e}} - T_{\text{w}}\right) \\ &= T_{\text{w}} + f_{\text{superheater}}\left(\frac{\eta_{\text{opt}}\dot{q}_{\text{solar}} + U_{\text{loss}}T_{\infty} + U_{\text{gain}}T_{\text{w}}}{U_{\text{loss}} + U_{\text{gain}}} - T_{w}\right) \end{aligned} \tag{25}$$

Supplementary Eq. (25) and the efficiency definition in Supplementary Eq. (16) both neglect the sensible heat of the steam above the saturation temperature. The efficiency could be modified to include the sensible heat

$$\eta = j\left[h_{\mathrm{g}}\left(T_{\mathrm{s}}\right) - h_{\mathrm{f}}\left(T_{\mathrm{ref}}\right)\right] \Big/ \dot{q}_{\mathrm{solar}} \tag{26}$$

where h_{f} is the specific enthalpy of the liquid and T_{ref} is a suitable reference temperature. Taking T_{ref} = 100 °C accounts for the latent heat of vaporization and the sensible heat from 100 °C to T_{s}. For the range of superheat temperatures achieved in this study, the efficiencies based on Supplementary Eq. (26) are maximally 4% (relative) higher than those based on Supplementary Eq. (17). Therefore, to maintain similarity with similar studies, we maintain Supplementary Eq. (17) as the definition for the efficiencies reported in this study.

Summary of parameters in steady-state model

Supplementary Eqs. (17) and (25) are the main equations of the steady-state model which can determine the efficiency and steam temperature as a function of the solar flux. Supplementary Table 1 gives a summary of the steady-state model parameters for the laboratory scale CSES, determined from a best-fit to the experimental data.

Supplementary Note 4. Optical efficiency

In this note, the optical efficiency of the system is calculated based on the optical properties measured in Supplementary Note 7.

The optical efficiency is comprised of three main components

$$\eta_{\text{opt}} = \tau_N \cdot \alpha_{\text{solar}} \cdot \text{AAF} = 0.864 \cdot 0.924 \cdot 0.95 = 75.8\% \tag{27}$$

where τ_N is the total solar transmittance of the three-layer glazing system (see Supplementary Note 6), α_{solar} is the solar absorptance of the selective surface, and AAF is the active area fraction, which accounts for the fact that some of the 15.24 × 15.24 cm absorber area is blocked by screws and screw gaskets. The active area fraction amounts to $f = 95\%$.

For the total solar transmittance of the FEP glazing system, we can assume that the internal transmittance the FEP layer is 100%, i.e. losses are only by Fresnel reflection at the interfaces. In this case, the overall transmittance (neglecting the interaction of the glazing system and the absorber) for a system of N layers is[4]

$$\tau_N = \frac{1 - \frac{1-\tau}{1+\tau}}{1 + (2N+1)\frac{1-\tau}{1+\tau}} \tag{28}$$

where τ is the reflectance of a single layer (roughly equal to τ_{solar} for a single FEP film). For $N = 3$ and $\tau = 0.951$ the total transmittance is 86.4%. Note that for high values of τ, the approximate equation

$$\tau_N \approx \tau^N \tag{29}$$

holds with good accuracy. Additionally, the error imposed by applying Supplementary Supplementary Eq. (28) directly to the solar weighted τ, rather than first applying it to τ_λ and then determining the solar weighted τ is small.

The solar weighted absorptance of the selective surface was determined to be 92.4%. This value will change slightly due to spectral changes imposed by the FEP stack. However, since the FEP transmittance is relatively spectrally flat, the error introduced by this approximation is small.

Combining, we find the overall optical efficiency to be 75.8%.

Supplementary Note 5. Transient numerical model

In this note, the transient numerical model for the CSES is explained. This model expands upon the steady-state model to include transient, nonlinear, and multidimensional effects which give more insight into the performance of the CSES. In particular, a transient model is necessary to simulate the performance of the CSES in outdoor conditions where the solar input is not constant.

The transient numerical model is a quasi-one-dimensional coupled heat and mass transfer model developed using the Simscape package in Matlab Simulink. Supplementary Fig. 4 gives the equivalent thermal circuit diagram for the model, with R representing thermal resistances and C representing thermal capacitances ($C = m \cdot c_\mathrm{p}$) of the main components of the device. The "Simulator Control", "SPS" and "Solver" blocks are under-the-hood controls and can be ignored for the purposes of understanding the physics of the model. The portions of the circuit pertaining to the side and bottom losses are outlined, with the remainder of the circuit describing the main path of heat flow from the absorber to the water reservoir. Multidimensional effects are accounted for through a distributed resistance arrangement, e.g. in the bottom loss and side loss sections. The "Water" and "Superheater" blocks are custom-made blocks described in detail below.

The governing equations for the model are as follows. For conduction and convection resistors

$$\frac{dQ}{dt} = \frac{T_\mathrm{A}(t) - T_\mathrm{B}(t)}{R} \tag{30}$$

where $R = 1/(UA)$ for convection and $R = L/(kA)$ for convection. For radiation resistors

$$\frac{dQ}{dt} = G\left(T_\mathrm{A}^4(t) - T_\mathrm{B}^4(t)\right) \tag{31}$$

where $G = \epsilon_\mathrm{eff} \sigma A$. For capacitances

$$\frac{dQ}{dt} = mc_\mathrm{p}\frac{dT}{dt} \tag{32}$$

To handle evaporation a custom-built "Water" block was programmed in the Simscape language in Matlab®. The "Water" component is described by the following governing equation

$$\begin{cases} \dfrac{dQ}{dt} = -h_{\text{fg}} \dfrac{dm_{\text{w}}}{dt}, \ \dfrac{dT_{\text{w}}}{dt} = 0 & \text{for } T_{\text{w}} = T_{\text{b}} \text{ or } m_{\text{w}} > 0 \\ \dfrac{dQ}{dt} = \left(m_{\text{w}} c_{\text{p,w}} + m_{\text{basin}} c_{\text{p,basin}} \right) \dfrac{dT_{\text{w}}}{dt}, \ \dfrac{dm_{\text{w}}}{dt} = 0 & \text{otherwise} \\ \dot{m} = \dfrac{dm_{\text{w}}}{dt} \end{cases} \tag{33}$$

where m_{w} is the mass of liquid water in the basin, m_{basin} is the mass of the basis itself, and c_{p} is the specific heat capacity, T_{b} is the boiling point of water, and $\dot{m}$ is the steam mass flow rate. The model neglects mass diffusion (see Supplementary Note 14) and therefore predicts a zero evaporation rate for $T < T_{\text{b}}$. Therefore, the small initial mass loss $\Delta m_{\text{heat-up}}$ during the heat-up phase (see Fig. 3 of the main paper) is not predicted by the transient model. A "Superheater" block was similarly custom-built following the governing equation

$$\frac{dQ}{dt} = f_{\text{superheater}} \dot{m} c_{\text{p,s}} \left(T_{\text{A}} - T_{\text{b}} \right) \tag{34}$$

where $f_{\text{superheater}}$ is the superheater effectiveness, $\dot{m} = dm_{\text{w}}/dt$ is the steam mass flow rate, and $c_{\text{p,s}}$ is the specific heat capacity of steam. The components are coupled by the conversation of energy at the nodes

$$\sum \frac{dQ}{dt} = 0 \tag{35}$$

The boundary conditions are the ambient temperature and the absorbed solar power input $\dot{Q}_{\text{abs}} = \eta_{\text{opt}} \dot{q}_{\text{solar}} A$. The initial condition comprises the initial temperature of all thermal capacitances (set equal to the ambient temperature) and the initial mass of water in the reservoir. The resulting set of ordinary differential equations (ODEs) was solved numerically using the Runge-Kutta technique with trapezoidal integration, as implemented by the ode23t solver in Simulink®. The simulation time for a single run is approximately 0.5 s.

The model parameters were chosen according to known material properties and geometries, and an informant multidimensional model discussed later in this note. The fact that the model agrees well with experiment without fitting is testament to the validity of the physical description of the heat transfer and evaporation process. The model parameters which need to be specified are the Rs, Gs, Cs for the various components, and the effectiveness $f_{\text{superheater}}$ for the superheater. The

parameters for a standard laboratory run are given in Supplementary Fig. 4. Specification of the most important parameters is discussed in the following.

Nominal mass flow rate

It is useful for the subsequent analysis to establish a nominal flow condition for the CSES. Taking an efficiency of 30% and a solar flux of 1,000 W m^{-2}, the nominal mass flux can be determined from

$$j = \frac{\eta \dot{q}_{\text{solar}}}{h_{\text{fg}}} = \frac{0.3 \cdot 1{,}000 \text{ W m}^{-2}}{2257 \text{ J g}^{-1}\text{K}^{-1}} = 0.133 \text{ g s}^{-1}\text{m}^{-2} \tag{36}$$

The corresponding nominal mass flow rate is

$$\dot{m} = jA = 0.133 \text{ g s}^{-1}\text{m}^{-2} \cdot \left(0.1524 \text{ m}\right)^2 = 3.1 \text{ mg s}^{-1} = 0.186 \text{ g min}^{-1} = 11.12 \text{ g hr}^{-1} \tag{37}$$

Emitter-water radiative exchange

Assume that the emitter, basin side walls, and water form a three-surface enclosure whose radiative exchange can be determined using the circuit analogy shown in Supplementary Fig. 5, where J is the radiosity, and e = emitter, and w = water.

Assuming that the sidewalls are perfectly insulated, the net radiative exchange between the emitter and the water may be determined from

$$\dot{Q}_{\text{rad}} = G\left(T_{\text{e}}^4 - T_{\text{w}}^4\right) = \frac{\sigma T_{\text{e}}^4 - T_{\text{w}}^4}{R_{\text{rad,tot}}} \tag{38}$$

From Supplementary Fig. 5

$$R_{\text{rad,tot}} = \frac{1-\epsilon_{\text{e}}}{A_{\text{e}}\epsilon_{\text{e}}} + \frac{1-\epsilon_{\text{w}}}{A_{\text{w}}\epsilon_{\text{w}}} + \left[\left(\frac{1}{A_{\text{e}}F_{\text{e-sidewall}}} + \frac{1}{A_{\text{w}}F_{\text{w-sidewall}}}\right)^{-1} + A_{\text{e}}F_{\text{e-w}}\right]^{-1} \tag{39}$$

Applying the enclosure and reciprocity relations for the view factors, Supplementary Eq. (39) simplifies to

$$R_{\text{rad,tot}} = \frac{1-\epsilon_{\text{e}}}{A_{\text{e}}\epsilon_{\text{e}}} + \frac{1-\epsilon_{\text{w}}}{A_{\text{w}}\epsilon_{\text{w}}} + \frac{A_{\text{e}} + A_{\text{w}} - 2A_{\text{e}}F_{\text{e-w}}}{A_{\text{e}}A_{\text{w}} - A_{\text{e}}^2F_{\text{e-w}}^2} \tag{40}$$

Treating the emitter and water surfaces as identical directly opposed squares, the view factor from the emitter to the water can be found from[5]

$$F_{\text{e-w}} = \frac{2}{\pi X^2}\left[\ln\left(\sqrt{\frac{(1+X^2)^2}{1+2X^2}}\right) + 2X\sqrt{1+X^2}\arctan\left(\frac{X}{\sqrt{1+X^2}}\right) - 2X\arctan X\right] \tag{41}$$

where X is the ratio of the side length of the square to separation between the squares. Taking $X = 14.9$ cm/1.5 cm as representative considering the average water height through the experiment, the emitter to water view factor is found to be $F_{\text{e-w}} = 0.813$. Taking this and values of $\epsilon_e = 0.941$, $\epsilon_w = 0.910$, $A_e = 0.0223$ m^2, $A_w = 0.0218$ m^2 and plugging in to Supplementary Eq. (40) gives $G/\sigma = 0.0176$ m^2. By inspection of Supplementary Eq. (38), the value $G/(\sigma A_e)$ can be interpreted as the effective emissivity of the system, which amounts to $\epsilon_{\text{eff}} = 0.790$. For modelling the experiments conducted with the radiation shield, the same methodology was used, except that ϵ_w was replaced with the effective emissivity from Supplementary Eq. (80).

Gas gap convection

Heat transfer through the gas gap is primarily by radiation, but conduction and convection are still present to some degree. As shown in Supplementary Note 9, the convective heat transfer through the gas gap can be represented by a heat transfer coefficient of 1.5 W m^{-2} K^{-1} and a thermal resistance of 30.58 K/W.

Sidewall conduction

In a large-scale system, conduction from the emitter to the water through the sidewall of the basin would be negligible due to the large area-to-perimeter ratio. In the smaller lab-scale device, the sidewall conduction is small, but non-negligible. The conduction thermal resistance is

$$R_{\text{cond}} = \frac{L_{\text{sidewall}}}{k_{\text{PEI}} A_{\text{sidewall}}} \tag{42}$$

where L_{sidewall} is the distance between the emitter and the water layer (1.5 cm), k_{PEI} is the thermal conductivity of the basin material (PEI) taken as 0.22 W m^{-1} K^{-1}, and A_{sidewall} is the effective conduction area of the sidewall. Due to the complex sidewall geometry (including the assembly screws), the thermal resistance was calculated using a 3D Finite Element Model similar to that described later in this section. The computed thermal resistance was 56.37 K/W.

Biot number and water temperature distribution

The Biot number for the water layer shows whether the water can be assumed isothermal. The maximum water depth, d_w, under normal conditions is 5 mm. The corresponding Biot number is

$$\mathrm{Bi} = \frac{U_{\mathrm{gain}}}{k_{\mathrm{w}}/d_{\mathrm{w}}} = \frac{12.8\ \mathrm{W\ m^{-2}K^{-1}}}{0.672\ \mathrm{W\ m^{-1}K^{-1}}/0.005\ \mathrm{m}} = 0.095 \tag{43}$$

Typically $\mathrm{Bi} < 0.1$ signifies that internal temperature gradients are small compared to the external gradient, i.e. that the water layer can effectively be treated as isothermal.

Superheater heat transfer

The function of the superheater is to transfer heat from the hot absorber/emitter to the saturated water vapour. To achieve good solid-to-gas heat transfer, a porous reticulated vitreous carbon (RVC) foam was selected (ERG Duocell® 100 PPI). The foam features a high specific surface area (SSA) of 6562 m^2/m^3 providing a large area for solid-gas heat exchange.

The superheater can be modeled as a gas-solid heat exchanger following the effectiveness vs. number of transfer units (f-NTU) methodology[3] (here we use the symbol f in place of the more common effectiveness symbol ε to avoid being confused with emissivity). Elaborating upon the formulation defined in Supplementary Note 3, the effectiveness of a heat exchanger is defined as

$$f_{\mathrm{superheater}} \equiv \frac{\dot{Q}}{\dot{Q}_{\mathrm{max}}} = \frac{\dot{Q}}{\dot{m}c_{\mathrm{p}}\left(T_{\mathrm{C,in}} - T_{\mathrm{H,in}}\right)} \tag{44}$$

where $\dot{Q}$ is the actual total rate of heat transfer in the heat exchanger, and $\dot{Q}_{\mathrm{max}}$ is the maximum possible rate of heat transfer which follows from thermodynamic considerations. In the general case $\dot{m}c_{\mathrm{p}}$ is evaluated for the fluid having the lowest heat capacitance rate, $\dot{C}_{\mathrm{min}} = \min(\dot{m}c_{\mathrm{p}})$, $T_{\mathrm{C,in}}$ is the coldest part of the heat exchanger (typically the inlet temperature of the colder fluid), and $T_{\mathrm{H,in}}$ is the hottest part of the heat exchanger (typically the inlet temperature of the hotter fluid). In this system there is only a single fluid, and we may take $T_{\mathrm{C,in}}$ as the temperature of the vapor at the inlet (saturation temperature) and $T_{\mathrm{H,in}}$ as the emitter temperature. Once the effectiveness of the heat exchanger is known, the actual rate of heat transfer to the steam can be determined from Supplementary Eq. (44).

To determine the effectiveness, the number of transfer units, NTU, a dimensionless quantity representing the available heat exchange area, is required. It is defined as

$$\mathrm{NTU} = \frac{UA}{\dot{m}c_{\mathrm{p,s}}} \tag{45}$$

where U is the overall heat transfer coefficient from the absorber/emitter to the steam, A is the gas-solid heat exchange area, $\dot{m}$ is the steam mass flow rate, and $c_{p,s}$ is the constant-pressure specific heat capacity of the steam.

Assuming a minimum Nusselt number of 2 for internal flow[6], and a nominal pore diameter of 254 μm (based on the nominal linear porosity of 100 pores per inch, approximately 4 pores per mm, with a very narrow pore size distribution) the minimum solid-gas heat transfer coefficient is estimated to be

$$U_{\text{solid-vapour,RVC}} = \frac{\text{Nu} \cdot k_{\text{s}}}{d_{\text{pore}}} = \frac{2 \cdot 0.025 \text{ W} \cdot \text{m}^{-1}\text{K}^{-1}}{254 \times 10^{-6}\text{m}} = 197 \text{ W} \cdot \text{m}^{-2}\text{K}^{-1} \quad (46)$$

For the overall heat transfer coefficient, we must also take into account the heat conduction resistance through the RVC, which is determined using the effective thermal conductivity of the RVC foam reported by the manufacturer, and assuming that, on average, heat must be conducted through half the thickness of the RVC foam. The resulting resistance is

$$R_{\text{cond,RVC}} = \frac{L_{\text{cond,RVC}}}{k_{\text{eff,RVC}} A_{\text{cond,RVC}}} = \frac{3.175 \text{ mm}}{0.05 \text{ W} \cdot \text{m}^{-1}\text{K}^{-1} \cdot 16{,}895 \text{ mm}^2} = 3.76 \text{ K/W} \quad (47)$$

The overall heat transfer coefficient area product is therefore

$$UA_{\text{RVC}} = \left(R_{\text{cond,RVC}} + \frac{1}{U_{\text{solid-vapour,RVC}} \cdot A_{\text{solid-vapour,RVC}}} \right)^{-1} \quad (48)$$

where

$$A_{\text{solid-vapour,RVC}} = V_{\text{RVC}} \cdot \text{SSA}_{\text{RVC}} = 107.28 \text{ cm}^3 \cdot 6562 \text{ m}^{-1} = 0.704 \text{ m}^2 \quad (49)$$

which yields

$$UA_{\text{RVC}} = \left(3.76 \text{ K/W} + 0.007 \text{ K/W}\right)^{-1} = 0.265 \text{ W/K} \quad (50)$$

Interestingly, the major contributor to the overall thermal resistance is the conduction through the foam which implies that the design could be improved by using a superheater heat exchange material with improved bulk thermal conductivity such as a graphitic or metallic foam.

Based on nominal mass flow rate, see Supplementary Eq. (37), of 0.0031 g/s the number of transfer units is

$$\mathrm{NTU} = \frac{UA_{\mathrm{RVC}}}{\dot{m}_{\mathrm{s}} c_{\mathrm{p,s}}} = \frac{0.265\ \mathrm{W/K}}{0.0031\ \mathrm{g/s} \cdot 2.029\ \mathrm{J \cdot g^{-1} K^{-1}}} = 42.1 \tag{51}$$

With NTU known, then effectiveness can be determined from standard correlations based on the heat exchanger geometry. Most heat exchanger correlations are developed for fluid-fluid heat exchangers, where both fluids undergo temperature changes as they flow through the heat exchanger. In our case, only the steam is being heated. As discussed in Supplementary Note 3, the sensible heat is a small fraction of the latent heat. Therefore, the emitter energy balance is dominated by heat transfer to the liquid water, i.e. that associated with evaporation, and heat transfer to the steam has only a small effect on the emitter temperature. Therefore, for the purposes of the heat exchanger analysis, we can assume that the emitter temperature remains constant regardless of the amount of heat delivered to superheat the steam. This implies that the emitter effectively has a heat capacitance rate $\dot{C}_{\mathrm{max}} \to \infty$, i.e. its temperature does not change as a result of transferring heat to the steam. This greatly simplifies the heat exchanger analysis because the performance of all heat exchangers with $\dot{C}_{\mathrm{min}}/\dot{C}_{\mathrm{max}} \to 0$ may be described by a single universal f-NTU correlation[3]:

$$f_{\mathrm{superheater}} = 1 - \exp\left(-\mathrm{NTU}\right) \tag{52}$$

Plugging in the value from Supplementary Eq. (51) into Supplementary Eq. (52) we find that the effectiveness of the superheater is approximately 100%, which is not surprising considering the very large heat exchange area of the RVC foam. However, the performance of the superheater in the real system is lower due to several practical considerations: 1) backwards flow and flow mixing which tends to reduce the local temperature difference and heat transfer rates; 2) non-uniform emitter temperature; and 3) vapour cooling in the outlet tube. Experimental observations indicate that these effects reduce the superheater effectiveness to a value of $f_{\mathrm{superheater}} = 0.8$, which was subsequently used for all calculations.

Multilayer polymer glazing

As seen in Supplementary Eqs. (17) and (15), achieving high efficiency and emitter (steam) temperature requires minimizing U_{loss}. This may be achieved through use of a spectrally selective surface to minimize radiation losses, and a glazing system to minimize convection losses.

The layer spacing was chosen to maximize thermal resistance. Thermal resistance increases linearly with layer spacing up to the critical spacing for the onset of natural convection, beyond which point the thermal resistance remains relatively constant with increased spacing. The critical spacing occurs when the Rayleigh number reaches its critical value[3]

$$\mathrm{Ra} = \mathrm{Gr}\,\mathrm{Pr} = \frac{g\beta\,\mathrm{Pr}}{\nu^2}\Delta T \cdot L_{\text{glazing gap}}^3 = 1,708 \tag{53}$$

The Rayleigh number is generally larger at lower mean temperatures, primarily due to β being large at low temperatures. Taking a three-layer stack with spacing 6.2 mm, assuming a 50 K temperature differential across the layer and conservatively evaluating the air properties at 300 K gives

$$\mathrm{Ra} = 400 < 1,708 \tag{54}$$

Therefore, we do not expect natural convection to occur in the multilayer glazing. This analysis is supported by no observation of fluttering of the thin films during operation, which would be a sign of natural convection currents. For the three-layer polymer glazing used in the final design, the heat transfer coefficient is

$$U_{\text{conv,glazing}} = \frac{k_{\text{air}}}{3L_{\text{glazing gap}}} = \frac{0.03\ \mathrm{W\ m^{-1}K^{-1}}}{3\cdot 6.2\ \mathrm{mm}} = 1.61\ \mathrm{W\ m^{-2}K^{-1}} \tag{55}$$

Radiation losses

Radiation losses were minimized by using a spectrally selective absorber. The optical properties of the absorber were measured as reported in Supplementary Note 7. Based on representative conditions an emittance of 0.081 was taken for the selective surface. The radiation losses are somewhat higher due to the fact that 4% of the absorber area is covered by polymer screws which we assume to have an emittance of 1. The effective emittance is therefore taken as

$$\epsilon_{\text{eff,top}} = 0.081\cdot 0.96 + 1\cdot 0.04 = 0.118 \tag{56}$$

Top, bottom, and side losses

Due to the aspect ratio (height to width ratio) of the laboratory device, conduction through the insulation cannot be treated as one-dimensional. Therefore, to inform the transient heat transfer model, a steady-state 3D heat conduction model (see Supplementary Note 16) was developed. The 3D conduction model allows the multidimensional effects to be simply incorporated into the

transient numerical model as top, bottom, and side losses with effective heat transfer coefficients of 1.66, 0.87, and 0.96 W $m^{-2}K^{-1}$, respectively

Other losses

The parasitic losses account for additional sources of loss, primarily due to heat leakage to the environment via conduction through the outlet tube.

Supplementary Note 6. Details of the CSES design

In this note, the salient design features of the laboratory scale CSES are discussed.

CSES device

Supplementary Fig. 6 shows an exploded view of the CSES. Several design iterations were constructed and tested before arriving at the final design shown here. The final device best demonstrated the two most important physical phenomena, namely contactless steam generation and superheating at one-sun. Working from the bottom of the schematic upwards, a basin was machined from polyetherimide (PEI), a high temperature polymer, chosen to limit thermal conduction to the water through the basin sidewall. Additionally, PEI's hydrophobicity prevents salt from climbing up the side of the basin wall. The basin can accommodate a maximum of approximately 400 g of water. 100 g of water was used for the laboratory experiments, 150 g was used for the outdoor experiment, and 50 g for the outdoor experiment with collection. The superheater shell was machined from 6061 aluminium alloy. It sits on top of the basin sidewall, and the interface is sealed using silicone gasket. The bottom of the superheater shell was painted with a high-emissivity paint Zynolyte® Hi-Temp, thus forming the emitter surface. A radiation shield constructed from aluminium foil was optionally placed between the emitter and the water to reduce the effective emissivity (see Supplementary Note 12) of the emitter surface allowing control of the emitter and superheated steam temperature. The degree of shielding was controlled by changing the size of the central hole in the radiation shield. We tested shields with two different hole sizes 5.08 cm × 5.08 cm and 7.62 cm × 7.62 cm corresponding to shield coverages (see Supplementary Note 12 and Supplementary Fig. 13) of $C_{\text{shield}} = 88\%$ and $C_{\text{shield}} = 73\%$ respectively. The superheater shell houses the reticulated vitreous carbon (RVC) foam (ERG Duocell® 100 PPI), which acts as an effective superheater, due to its high specific surface area which assures good solid-vapour heat exchange. Generated vapour leaves the basin volume and enters the superheater shell through 12 discrete vapour holes near the edge of the superheater shell. This flow arrangement forces the saturated steam to flow laterally through the RVC foam towards its centre, thus maximizing the residence time and promoting good solid-vapour heat exchange. This flow arrangement also forces the steam to exit through the hottest part of the system (the centre) thus maximizing the degree of superheat. The superheated steam exits through a single outlet tube, inserted into the superheater shell. A custom-built radiation-shielded thermocouple, detailed in

Supplementary Note 10, was inserted into the outlet tube to measure the temperature of the superheated vapour. The selective surface (Alanod eta plus®) was attached to a 6061 aluminium substrate using a thin layer of silicone adhesive. A silicone gasket was used to seal the interface between the bottom of the aluminium substrate and the top of the RVC foam and the edge of the superheater shell. The entire stack was assembled using 12 polyether ether ketone (PEEK) assembly screws. PEEK was chosen to minimize heat conduction from the absorber to the water. Leaks through the screw holes were sealed by silicone washers.

Multilayer polymer glazing

To minimize conduction/convection heat losses from the top of the CSES device, a multilayer polymer glazing system was developed. In general, a glazing system comprises a layer of one or more transparent sheets, separated by a gap of near-stationary air. The optimal number of layers is a trade-off between solar transmittance and thermal resistance. Traditional glazing systems use bulky and expensive glass sheets. Polymers can achieve significantly higher transmittance than glass layers owing to their lower index of refraction, and have the potential for lower cost and flexibility. We selected fluorinated ethylene propylene (FEP) as the transparent polymer due to its extremely high solar-weighted transmittance of over 95% for a single layer (see Supplementary Note 7), chemical stability, and ultraviolet resistance. Each layer is comprised of a stack of three materials: an aerogel felt (Aspen Aerogels® Pyrogel® 2250 DS) Pyrogel® sheet on the bottom, followed up a semi-rigid polyimide foam (Pyropel MD12) board, to which a 50 μm thick FEP polymer (Toray Advanced Film Co. TOYOFLON™) layer is affixed, as seen in Supplementary Fig. 7. Pyropel was chosen due to its combination of low thermal conductivity (0.036 W $m^{-2}K^{-1}$ at room temperature) and its reasonably rigid construction which makes it a good support material for the FEP film. Pyrogel® was chosen as an intermediate layer due to its ultra-low thermal conductivity (0.015 W $m^{-2}K^{-1}$ at room temperature). The triple-layer polymer glazing achieves an overall solar transmittance of 86.4% and an effective heat transfer coefficient of 1.6 W $m^{-2}K^{-1}$ (see Supplementary Note 5). Considering this in series with a convective heat transfer coefficient of 5 W $m^{-2}K^{-1}$, the overall heat transfer coefficient for the cover amounts to 1.2 W $m^{-2}K^{-1}$.

Experimental assembly

The CSES device was placed in an insulating box to minimize heat losses from the bottom and sides as shown in Supplementary Fig. 7. The CSES device is clad with several layers of aerogel

felt on the bottom and sides, followed by several layers of rigid polyisocyanurate insulation (Dow TUFF-R™, thermal conductivity 0.027 W $m^{-2}K^{-1}$ at room temperature).

Supplementary Note 7. Optical properties measurement

This note describes the measurement procedure and results of the optical properties of the materials used in the CSES.

Optical properties of relevant materials were measured over a broad wavelength range 250 nm to 25 μm by Ultraviolet-Visible-Near-infrared (UV-Vis-NIR) and Fourier Transform Infrared (FTIR) spectroscopy, using an Agilent Cary 5000 spectrophotometer, and Thermo Fisher Nicolet 6700 Fourier transform infrared spectrometer, respectively. Supplementary Table 2 shows a summary of the most important optical properties for the materials used in the device. All measurements were performed at room temperature. Values reported at elevated temperatures were obtained by averaging the spectral properties by the Planck spectrum at the given temperature.

Selective surface

Solar absorptance was determined from standard 8°/hemispherical reflectance measurements performed using a UV-Vis-NIR integrating sphere coupled to the UV-Vis-NIR spectrometer. The reflectance factor was measured relative to a calibrated Spectralon® diffuse reference standard (Labsphere SRS-99-020) using the substitution method, and the absolute reflectance was subsequently determined. Absorptance in the infrared (thermal emittance) was determined from standard 8°/hemispherical reflectance measurements using a diffuse gold integrating sphere coupled to the FTIR spectrometer. The absorptance (emittance) spectrum for the selective surface is shown in Supplementary Fig. 8a.

The solar-weighted absorptance α_{solar} was determined using the averaging procedure of Supplementary Eq. (57) but replacing τ_λ by $\alpha_\lambda = (1 - \rho_\lambda)$ in the integral. Similarly the thermal emittance $\epsilon(T)$ was determined using the averaging procedure of Supplementary Eq. (58) but again replacing τ_λ by $\epsilon_\lambda = (1 - \rho_\lambda)$ in the integral. The resulting total emittance as a function of temperature is given in Supplementary Fig. 8b.

Advanced FEP glazing material

Fluorinated ethylene propylene (FEP, trade name Toyoflon™) films (thickness 50 μm) were obtained from Toray Advanced Film Co., Ltd. The direct (normal incidence) transmission spectra of the film is given in Supplementary Fig. 9.

The solar transmittance can be computed from

$$\tau_{\text{solar}} = \frac{\int_{0.25\ \mu\text{m}}^{4\ \mu\text{m}} \tau_\lambda \dot{q}_{\text{solar},\lambda} d\lambda}{\dot{q}_{\text{solar}}} \tag{57}$$

where $\dot{q}_{\text{solar},\lambda}$ is the spectral solar irradiance following the global air mass 1.5 (AM1.5G) distribution[11], and $\dot{q}_{\text{solar}}$ is the total AM1.5G solar irradiance, which integrates to 1000.4 W m^{-2}. Similarly, the infrared transmittance can be determined from

$$\tau_{\text{IR}}\left(T_{\text{bb}}\right) = \frac{\int_{0.25\ \mu\text{m}}^{100\ \mu\text{m}} \tau_\lambda \dot{q}_{\text{bb},\lambda}\left(T_{\text{bb}}\right) d\lambda}{\sigma T_{\text{bb}}^4} \tag{58}$$

where $\dot{q}_{\text{bb},\lambda}(T_{\text{bb}})$ is the hemispherical spectral emissive power for a blackbody source at temperature T_{bb} obtained from the Planck distribution. The resulting solar and infrared transmittance are given in Supplementary Table 2.

Emitter

The emitter was coated with Zynolyte® Hi-Temp aerosol spray paint in five even layers. The paint was dried at room temperature for 30 minutes and then cured by twice cycling from room temperature to 250 °C on a hot plate, resulting in a uniform diffuse black coating. The emittance was determined by measuring the infrared hemispherical reflectance spectrum via FTIR. The spectral emittance was relatively constant with a minimum and maximum value of 90% and 97% over the range 2.5 μm to 20 μm. As a result, the total emittance of the emitter is nearly invariant with temperature, with a value of $\epsilon_e = 0.941 \pm 1\%$ over the range 0 °C to 400 °C.

Supplementary Note 8. Reducing transient heat-up time

Due to the diurnal and intermittent nature of sunlight, it is beneficial to reduce the transient heat-up time of the CSES. The validated transient numerical model was used to determine the effect of both the thermal mass of the components of the CSES and the initial depth of water in the basin on the transient response of the system. Supplementary Fig. 10 shows the transient response of the system for four cases: 1) the as-tested CSES design; 2) an optimized CSES design where the thermal mass of the components has been reduced by 75%; 3) the as-tested CSES design where the mass (depth) of water initially filled in the basin is reduced by 75%; and 4) the case where both reductions have been made.

Supplementary Note 9. Convection in the gas gap

A rectangular cavity with heated top surface is inherently stable against buoyancy driven flows, i.e. natural convection, therefore we do not expect to have natural convection currents above the water reservoir inside the basin. However, there will be a flow caused by the heat-transfer driven evaporation. This upward flow from the water basin through the superheater will affect the temperature distribution in the gas gap, and thusly the conduction through the gas gap. In this analysis, we determine the effect of this upward evaporative flow on the conduction from the superheater to the water through the gas gap.

Consider a generic control volume *Adz* in the gas gap as shown schematically in Supplementary Fig. 11. Assume that evaporation generates a one-dimensional vapour flow rate upward through the gas gap.

An energy balance on the control volume gives

$$-\dot{m}c_{\mathrm{p,s}}\frac{dT}{dz}+k_{\mathrm{s}}A\frac{d^2T}{dz^2}=0 \tag{59}$$

This can be nondimensionalized using the Péclet number

$$\mathrm{Pe}=\frac{u}{\alpha_{\mathrm{s}}}L_{\mathrm{gas\ gap}}=\frac{\dot{m}/(\rho_{\mathrm{s}}A)}{k_{\mathrm{s}}/(\rho_{\mathrm{s}}c_{\mathrm{p,s}})}L_{\mathrm{gas\ gap}}=\frac{jc_{\mathrm{p,s}}L_{\mathrm{gas\ gap}}}{k_{\mathrm{s}}} \tag{60}$$

where u is the steam velocity, α_{s} is the steam thermal diffusivity, ρ_{s} is the steam density, k_{s} is the thermal conductivity of steam, $c_{\mathrm{p,s}}$ is the constant pressure specific heat of steam, $\dot{m}$ is the steam mass flow rate, j is the mass flux (per unit area), A is the area, and $L_{\mathrm{gas\ gap}}$ is the gas gap size. A Péclet number larger than unity indicates heat transfer dominated by bulk fluid motion, a Péclet number smaller than one indicates heat transfer dominated by stationary conduction, and a Péclet number near one indicates both mechanisms are important. Subbing in to Supplementary Eq. (59) gives

$$-\frac{\mathrm{Pe}}{L_{\mathrm{gas\ gap}}}\frac{dT}{dz}+\frac{d^2T}{dz^2}=0 \tag{61}$$

Twice integrating and solving for T gives

$$T = C_1 + C_2 \exp\left(\mathrm{Pe}\frac{z}{L_{\text{gas gap}}}\right) \tag{62}$$

The boundary conditions are as follows. Let $z = 0$ be the top surface of the water, then $T(z = 0) = T_{\mathrm{w}}$. For the upper boundary condition at $z = L_{\text{gas gap}}$, assume that the steam is at the emitter temperature T_{e}. In the end we are interested in the heat transfer to the water, which is related to the local derivative at $z = 0$. Therefore we do not expect our results to be very sensitive to the choice of $T(z = L_{\text{gas gap}})$. Inserting these boundary conditions gives

$$T(z) = T_{\mathrm{w}} + \frac{\exp\left(\mathrm{Pe}\frac{z}{L_{\text{gas gap}}}\right) - 1}{\exp(\mathrm{Pe}) - 1}\left(T_{\mathrm{e}} - T_{\mathrm{w}}\right) \tag{63}$$

With the temperature profile known, the heat transfer to the water can be determined from

$$\dot{q}_{\mathrm{w}} = k_{\mathrm{s}} \left.\frac{dT}{dz}\right|_{z=0} = k_{\mathrm{s}} \frac{T_{\mathrm{e}} - T_{\mathrm{w}}}{L_{\text{gas gap}}} \frac{\mathrm{Pe}}{\exp(\mathrm{Pe}) - 1} \tag{64}$$

For $\mathrm{Pe} \to 0$, the coefficient $\mathrm{Pe}/(\exp(\mathrm{Pe}) - 1) \to 1$, and Supplementary Eq. (64) simplifies to pure conduction, and for $\mathrm{Pe} \to \infty$, $\mathrm{Pe}/(\exp(\mathrm{Pe}) - 1) \to 0$ such that there is no nonradiative heat transfer to the water through the gas gap.

Under representative conditions (nominal mass flow rate and steam properties at 373 K), the Péclet number is

$$\mathrm{Pe} = \frac{0.133\ \mathrm{g\ s^{-1} m^{-2}} \cdot 2.079\ \mathrm{J\ g^{-1} K^{-1}} \cdot 0.015\ \mathrm{m}}{0.025\ \mathrm{W\ m^{-1} K^{-1}}} = 0.166 \tag{65}$$

The coefficient is

$$\frac{\mathrm{Pe}}{\exp(\mathrm{Pe}) - 1} = 0.92 \tag{66}$$

Therefore, the gas gap conduction is slightly less than the pure conduction case that would take place if the gas gap was stationary. For representative conditions, the gas gap heat transfer coefficient is

$$U_{\text{conv,gas gap}} = \frac{\mathrm{Pe}}{\exp(\mathrm{Pe}) - 1} \frac{k_{\mathrm{s}}}{L_{\text{gas gap}}} = 0.92 \frac{0.025\ \mathrm{W\ m^{-1} K^{-1}}}{0.015\ \mathrm{m}} = 1.5\ \mathrm{W\ m^{-2} K^{-1}} \tag{67}$$

Supplementary Note 10. Radiation-shielded thermocouple

This note discusses the design and performance of the radiation-shielded thermocouple designed and constructed to accurately measure the steam temperature at the outlet of the CSES.

Thermocouple measurements of gas temperatures in radiating environments must be performed with caution. For this purpose, a custom-built radiation-shielded thermocouple was constructed. Supplementary Fig. 12 shows a schematic of the radiation-shielded thermocouple assembly inside the CSES outlet tube.

A steady-state heat flux balance on the thermocouple tip gives

$$\begin{aligned} 0 &= \dot{q}_{\mathrm{conv}} + \dot{q}_{\mathrm{cond}} + \dot{q}_{\mathrm{rad}} \\ &= U_{\mathrm{conv,TC}}\left(T_{\mathrm{s}} - T_{\mathrm{TC}}\right) + U_{\mathrm{cond,TC}}\left(T_{\mathrm{e}} - T_{\mathrm{TC}}\right) + U_{\mathrm{rad,TC}}\left(T_{\mathrm{e}} - T_{\mathrm{TC}}\right) \end{aligned} \tag{68}$$

Assuming the linearized form of the radiation heat transfer coefficient, Supplementary Eq. (68) can be solved for the thermocouple junction temperature:

$$T_{\mathrm{TC}} = \frac{U_{\mathrm{conv,TC}}}{U_{\mathrm{conv,TC}} + U_{\mathrm{cond,TC}} + U_{\mathrm{rad,TC}}} T_{\mathrm{s}} + \frac{U_{\mathrm{cond,TC}} + U_{\mathrm{rad,TC}}}{U_{\mathrm{conv,TC}} + U_{\mathrm{cond,TC}} + U_{\mathrm{rad,TC}}} T_{\mathrm{e}} \tag{69}$$

The radiation error can be minimized by minimizing $U_{\mathrm{cond,TC}}$ and $U_{\mathrm{rad,TC}}$, while maximizing $U_{\mathrm{conv,TC}}$. To determine the convection heat transfer coefficient, we need the Reynolds number of the steam flow over the thermocouple junction. Based on the nominal mass flow rate, the velocity of the steam passing over the thermocouple is

$$u = \frac{4\dot{m}}{\rho_{\mathrm{s}}\pi D_{\mathrm{TC}}^{2}} = \frac{4\cdot 0.0031\times10^{-3}\ \mathrm{kg\ s^{-1}}}{0.55\ \mathrm{kg\ m^{-3}}\cdot\pi\cdot\left(3.25\times10^{-3}\ \mathrm{m}\right)^{2}} = 0.68\ \mathrm{m\ s^{-1}} \tag{70}$$

Note that this is the average velocity across D. The velocity in the centre of the tube where the thermocouple is located is likely higher. The Reynolds number over the thermocouple tip is

$$\mathrm{Re} = \frac{\rho_{\mathrm{s}} u d_{\mathrm{TC}}}{\mu_{\mathrm{s}}} = \frac{0.55\ \mathrm{kg\ m^{-3}}\cdot 0.68\ \mathrm{m\ s^{-1}}\cdot 0.2\times10^{-3}\ \mathrm{m}}{1.34\times10^{-5}\ \mathrm{Pa\ s}} = 5.6 \tag{71}$$

Using the Nusselt correlation from Whitaker[12], and taking Pr = 1

$$\mathrm{Nu} = 2 + \left(0.4\,\mathrm{Re}^{1/2} + 0.06\,\mathrm{Re}^{2/3}\right) = 2 + \left(0.4\cdot 5.6^{1/2} + 0.06\cdot 5.6^{2/3}\right) = 3.14 \tag{72}$$

The convective heat transfer coefficient is thus

$$U_{\text{conv,TC}} = \frac{\text{Nu} \cdot k_{\text{s}}}{d_{\text{TC}}} = \frac{3.14 \cdot 0.025 \text{ W m}^{-1}\text{K}^{-1}}{0.2 \times 10^{-3} \text{ m}} = 392 \text{ W m}^{-2}\text{K}^{-1} \tag{73}$$

The conduction heat transfer term accounts for conduction heat transfer from the surroundings to the thermocouple tip through the solid material. The main resistance to conduction is through the length L_{TC} of thermocouple wire. The resistance is

$$R_{\text{cond,TC}} = \frac{L_{\text{TC}}}{k_{\text{TC}} \cdot A_{\text{wire cross-section}}} = \frac{5 \times 10^{-3} \text{ m}}{30 \text{ W m}^{-1}\text{K}^{-1} \cdot 2 \cdot 0.005 \text{ mm}^2} = 3.33 \times 10^6 \text{ K W}^{-1} \tag{74}$$

To determine the effective heat transfer coefficient, we need to normalize to the heat transfer area of the thermocouple tip

$$\begin{aligned}
\dot{q}_{\text{cond}} &= \frac{\dot{Q}_{\text{cond}}}{A_{\text{TC}}} = \frac{\Delta T}{R_{\text{cond,TC}} A_{\text{TC}}} = h_{\text{cond}} \Delta T \\
U_{\text{cond,TC}} &= \frac{1}{R_{\text{cond,TC}} A_{\text{TC}}} = \frac{1}{R_{\text{cond}} \cdot \pi d^2} \\
&= \frac{1}{3.33 \times 10^6 \text{ K W}^{-1} \cdot \pi (0.2 \times 10^{-3} \text{ m})^2} = 2.39 \text{ W m}^{-2}\text{K}^{-1}
\end{aligned} \tag{75}$$

In the worst case for radiation, the surroundings can be modelled as a blackbody. In this case, a conservative (high) estimate of the radiation heat transfer coefficient is

$$U_{\text{rad,TC}} = 4 \epsilon_{\text{TC}} \sigma T_{\text{m}}^3 = 4 \cdot 0.1 \cdot \sigma (423 \text{ K})^3 = 1.71 \text{ W m}^{-2}\text{K}^{-1} \tag{76}$$

where ϵ_{TC} is the emissivity of the thermocouple tip (chromel-alumel)[13] taken as 0.1. Plugging in representative values we find a worst-case estimate for the thermocouple tip temperature

$$T_{\text{TC}} = 0.99 T_{\text{s}} + 0.01 T_{\text{e}} \tag{77}$$

In reality, the shield will be at a temperature in between T_{s} and T_{e}, further reducing the radiation error. Therefore, we conclude that the radiation error is below 1%.

Supplementary Note 11. Details of efficiency calculation

The steady-state efficiency was calculated according to

$$\eta = \frac{j}{\dot{q}_{\text{solar}}} = \frac{\Delta m_{\text{ss}}}{\dot{q}_{\text{solar}} A \Delta t_{\text{ss}}} \tag{78}$$

where Δm_{ss} is the evaporated mass during the steady state region and Δt_{ss} is the duration of the steady state region. The evaporated mass was determined by measuring the mass of the water in the basin before and after the experiment Δm_{basin} and subtracting the mass of water that was evaporated from the basin during the heat-up phase $\Delta m_{\text{basin,heat-up}}$

$$\Delta m_{\text{ss}} = \Delta m_{\text{basin}} - \Delta m_{\text{basin,heat-up}} \tag{79}$$

This approach is more reliable than an approach based on the instantaneous mass reading from the balance due to balance drift, vapour desorption from porous insulation materials, and leaks, which can affect the instantaneous reading but do not affect the overall mass change of the water in the basin. The evaporated mass during heat-up was determined from the balance measurement, e.g. from the mass curve shown in Fig. 3 in the main paper. The total mass loss during the heat-up phase is attributed to both evaporation of water in the basin, and evaporation of residual water and moisture in the system. Based on our experimental observations, it was assumed that the mass loss during the heat-up phase is 50% due to evaporation of water in the basin, and 50% due to residual moisture. The error bars in Fig. 4a in the main paper are based on 0% residual mass loss and 100% residual mass loss respectively, and thus represent a rigorous bound on the efficiency.

Supplementary Note 12. Controlling the degree of superheat

Inspection of Supplementary Eq. (25) suggests that it should be possible to obtain higher steam temperatures be *decreasing* the gain heat transfer coefficient. This can be achieved by placing a radiation shield between the emitter and the water surface as shown schematically in Supplementary Fig. 13. The radiation shield is essentially a reflective layer with a single central hole of area A_h, or an array of distributed holes will total area A_h. The radiation shield effectively reduces the view factor from the emitter to the water, reducing the radiation heat transfer, and thus reducing the gain heat transfer coefficient.

Assume that the shield is made of a reflective material such that the net radiation heat flux to the shield is small compared to the conduction/convection from the surrounding steam. In this case, the shield temperature will be approximately equal to the water (steam) temperature, simplifying the analysis significantly. In this case, the radiative exchange can be approximated by Supplementary Eq. (12) by replacing ϵ_w by the area-averaged emittance of the water and shield material.

$$\epsilon_{w,eff} = \left(1 - C_{shield}\right)\epsilon_w + C\epsilon_{sh} \tag{80}$$

where $C_{shield} = 1 - A_h/A$ is the shield coverage ratio.

An important advantage of the radiative mode of heat transfer, in comparison to conduction or convection, is that the gain heat transfer coefficient can be easily controlled through radiative shielding. For example, a simple iris diaphragm or Venetian blind type shutter could be used to actively control C_{shield}, thus controlling the gain heat transfer coefficient and steam temperature. This sort of active control is advantageous for any applications, where it is necessary to deliver the steam at some minimum useful superheated temperature. By actively controlling the gain heat transfer coefficient it is possible to maintain a constant T_s even during periods of low and fluctuating insolation (e.g. cloudy periods, morning, and evening).

Supplementary Note 13. Details of the outdoor experiments with stationary concentrator

Outdoor experiments were performed in October 2017 on a rooftop site on MIT's main campus in Cambridge, Massachusetts. On a clear day in October, the peak global horizontal irradiance (GHI) in Cambridge, Massachusetts is approximately 600 W m^{-2} at solar noon. To augment the solar flux to levels more relevant for the CSES operation, a low-cost stationary solar concentrator was designed and constructed.

Supplementary Fig. 14 shows a photograph of the fabricated optical concentrator. The concentrator is of the Asymmetric Compound Parabolic Concentrator (ACPC) type[8], and features a large acceptance angle such that diurnal tracking is not required; only seasonal adjustment is necessary. The optic was fabricated from a laser-cut thin PMMA sheet, onto which a mirror film was affixed. The geometric concentration ratio of the ACPC is defined as

$$C_{\mathrm{g}} = a_{\mathrm{i}} / a_{\mathrm{o}} \tag{81}$$

where a_{i} is the inlet aperture width and a_{o} is the outlet aperture width, where the CSES device is placed. The theoretical maximum concentration ratio is $1/\sin\theta_{\mathrm{i}}$ where θ_{i} is the acceptance angle. To reduce the overall size of the optic, the ACPC was asymmetrically truncated, at the expense of a slight reduction in the concentration ratio. The final design achieves a geometric concentration ratio of 3.1. The actual flux at the exit of the concentrator may be determined from

$$\dot{q}_{\mathrm{o}} \approx \mathrm{DNI} \cdot \rho_{\mathrm{mirror}} \cdot C_{\mathrm{g}} \cdot \cos\vartheta + \mathrm{dHI} \tag{82}$$

where DNI is the direct normal solar irradiance, ρ_{mirror} is the solar weighted reflectance of the mirror material (0.80), and ϑ is the angle of incidence defined in Supplementary Fig. 14, and dHI is the diffuse horizontal solar irradiance. Global horizontal solar irradiance (GHI) data was available from a HOBO U30 Weather Station beside the experimental setup on the roof, operated by the MIT Sustainable Design Lab. The DNI and DHI values were estimated from the GHI using a modified version of the correlation of Orgill and Hollands[9]. The angle of incidence was computed using the sun position calculated from the NREL Solar Position Algorithm (SPA)[10]. Based on these calculations, the peak outlet flux reached during the experiment was 1,731 W m^{-2}.

Supplementary Note 14. Evaporation mechanism

This note discusses the evaporation mechanism in the CSES, specifically addressing the relative importance of mass diffusion resistance, flow resistance, and interface limitations in the evaporation process in the CSES. It is demonstrated that evaporation is heat transfer limited, and occurs by induced pressure driven flow activated when the water temperature reaches its normal boiling point.

Mass diffusion resistance

Traditional solar stills rely on mass transfer driven by a water vapour concentration gradient (which may be augmented by bulk fluid motion). We would like to estimate the role of mass transfer in the CSES. During a typical laboratory experiment, the CSES is assembled after filling the basin with approximately 5 mm water. At the start of the experiment, we expect that the remaining volume of the basin, the gas gap (approximately 15 mm in height), is filled with laboratory air. During the start of the transient phase, as the water is heated by the emitter, we expect the gas gap will quickly become saturated with water vapour. Once the gas gap becomes saturated with water vapour, the main mass transfer resistance is diffusion through the RVC and through the column of vapour in the outlet tube. To determine the upper limit on the mass transfer rate due to diffusion, we will neglect the resistance of the RVC, and consider only the mass transfer resistance of the outlet tube. In this simplifying case, the mass flow rate due to mass diffusion through the outlet tube is given by[3]

$$\dot{m} = \frac{C_{\text{v,inside}} - C_{\text{v},\infty}}{R_{\text{mass transfer}}} = A_{\text{tube}} M_{\text{w}} D_{\text{v-air}} \frac{C_{\text{v,inside}} - C_{\text{v},\infty}}{L_{\text{tube}}} \tag{83}$$

where M_{w} is the molar mass of water, $D_{\text{v-air}}$ is the binary diffusion coefficient of air and water vapour, and C_{v} is the molar concentration of water vapour. An upper limit on the vapour concentration inside during the steady-state region is

$$C_{\text{v,inside}} = \frac{p_{\text{inside}}}{R_{\text{u}} T_{\text{v}}} = \frac{101\ 325\ \text{Pa}}{8.314\ \text{J} \cdot \text{mol}^{-1}\text{K}^{-1} \cdot 373.15\ \text{K}} = 32.7\ \text{mol/m}^3 \tag{84}$$

A representative value for the vapour concentration outside is

$$C_{\text{v},\infty} = \frac{\phi p_{\text{sat}}}{R_{\text{u}} T} = \frac{0.3 \cdot 3.169\ \text{Pa}}{8.314\ \text{J} \cdot \text{mol}^{-1}\text{K}^{-1} \cdot 298\ \text{K}} = 3.84 \times 10^{-4}\ \text{mol/m}^3 \tag{85}$$

where ϕ is the relative humidity of air in the lab, taken as 0.3. The binary diffusion coefficient is given by the empirical correlation[3]

$$D_{\text{v-air}} = 1.87\times10^{-10}\frac{(T/\text{K})^{2.072}}{(p/\text{atm})} \tag{86}$$

which equates to 4.61×10^{-5} m^2/s at a representative temperature of 400 K. Taking the length of the tube as 0.1 m and an inner diameter of 0.56 cm, the mass flow rate due to diffusion becomes

$$\dot{m} = \frac{\pi\cdot(0.56\times10^{-2}\text{ m})^2}{4} 18\text{ g/mol}\cdot 4.61\times10^{-5}\text{m}^2/\text{s}\cdot\frac{30.5\text{ mol/m}^3}{0.1\text{ m}} = 0.02\text{ g/hr} \tag{87}$$

which is more than two orders of magnitude smaller than the flow rates exhibited in the CSES during the quasi-steady phase. We can therefore conclude that diffusion is not an important vapour generation mechanism.

Flow resistance

Instead of diffusion-dominated vapour generation, we posit a pressure-driven flow that dominates the vapour generation mechanism, which originates from a slight superheating (<< 1 K) at the water-vapour interface. When the water reservoir reaches its boiling point, this opens up a new channel for evaporation to occur. At the start of the steady-state region, the water is at 100 °C and the vapour at the interface can be assumed to be saturated vapour at 1 atm. When additional heat is transferred to the water, it will evaporate, since it is already at its saturation temperature at 1 atm. If the system was completely sealed, this generated vapour would increase the pressure of the system, which in turn would increase the saturation temperature (boiling point). The pressure and temperature would then both rise as heat is continually supplied and more liquid water is converted to vapour. If the closed system was then opened to the ambient, e.g. by way of a valve, the pressure difference would induce a flow of vapour. In the limit of no flow restriction, the pressure would be equal everywhere to the ambient pressure, and any net heat added to the water above its boiling point will go towards evaporation. With a partial flow restriction, the pressure at the liquid-vapour interface would be somewhat higher than the ambient pressure.

We measured the flow resistance by flowing air with a known flow rate through the outlet tube and measuring the pressure drop from the outlet tube to the basin, which was vented to ambient. Supplementary Fig. 16 shows the resulting pressure drop through the system as a function of the volumetric flow rate.

Under nominal conditions, the volumetric flow rate is

$$\dot{V} = \frac{\dot{m}}{\rho} = \frac{0.186 \text{ g min}^{-1}}{0.52326 \text{ g/L}} = 0.36 \text{ L min}^{-1} \tag{88}$$

From Supplementary Fig. 16 we can conclude that the pressure drop under nominal conditions is below 200 Pa. The change in saturation temperature due to this increase in pressure is given by the Clapeyron equation

$$\frac{\Delta T_{\text{sat}}}{\Delta p_{\text{sat}}} = \frac{RT^2}{M_{\text{w}} p_{\text{sat}} h_{\text{fg}}} = \frac{8.314 \text{ kJ kmol}^{-1}\text{K}^{-1} \cdot (373 \text{ K})^2}{18 \text{ kg/kmol} \cdot 101\,325 \text{ Pa} \cdot 2257 \text{ kJ/kg}} = 0.28 \text{ K/kPa} \tag{89}$$

For a 200 Pa pressure rise inside the reservoir, the change in the boiling point is just 0.06 K. This indicates that the flow resistance is very small, and the water temperature is essentially pinned at 100°C. In essense, when the temperature of the water T_{w} is raised by a small amount, p_{sat} grows exponentially, opening up the pressure driven flow pathway.

We can therefore conclude that the interface and flow resistances are very small, and the mass diffusion resistance is very large. This indicates that evaporation in the CSES is driven by exponential growth of p_{sat} when the water temperature is raised above its normal boiling point, which leads to a pressure driven flow enabled by the small flow resistance.

Interface resistance

Comparison of the mass diffusion and flow resistances indicated that the dominant mechanism is pressure driven flow. The interface resistance is given implicitly by the Hertz-Knudsen equation[7]

$$\dot{m} = AM_{\text{w}} \sqrt{\frac{M_{\text{w}}}{2\pi R_{\text{u}}}} \left(\sigma_{\text{e}} \frac{p_{\text{sat}}(T_{\text{w}})}{\sqrt{T_{\text{w}}}} - \sigma_{\text{c}} \frac{p_{\text{v}}}{\sqrt{T_{\text{v}}}} \right) \tag{90}$$

where T_{w} and T_{v} are the temperatures of the water and vapour just below and above the interface, respectively, $p_{\text{sat}}(T_{\text{w}})$ is the saturation pressure at T_{w}, and p_{v} is the vapour partial pressure just above the interface, and σ_{e} and σ_{c} are the evaporation and coefficients. For simplicity, we assume $\sigma_{\text{e}} = \sigma_{\text{c}} = 1$ and $T_{\text{w}} = T_{\text{v}}$ in which case Supplementary Eq. (90) simplifies to

$$\dot{m} = AM_{\text{w}} \sqrt{\frac{M_{\text{w}}}{2\pi R_{\text{u}} T_{\text{w}}}} \Delta p \tag{91}$$

where Δp is the pressure difference across the interface which drives evaporation. From Supplementary Eq. (91) we can calculate the required Δp for the nominal mass flow rate in the CSES given by Supplementary Eq. (37), which amounts to 8 Pa. We can therefore conclude that the interface resistance is small, again pointing to a heat-transfer limited evaporation mechanism.

Supplementary Note 15. Details of the laboratory experiments

This note provides additional details of the experimental setup and procedures not included in the Methods section of the main paper.

Supplementary Fig. 17 shows the laboratory experimental setup. A beam of simulated solar radiation leaves the Class AAA solar simulator (ScienceTech, SS-1.6K), and is directed downward to the CSES by a beam-down mirror. The solar simulator nominally outputs a flux of 1,000 W m^{-2}. Due to the slightly divergent beam exiting the simulator, the flux can be adjusted by moving the beam-down mirror/CSES assembly toward/away from the simulator outlet to increase/decrease the flux. The flux can then be fine-tuned by adjusting the current delivered to the lamp. Using both techniques, the flux was varied between 500 and 1,800 W m^{-2} over the course of the experiments.

A standard laboratory experiment consisted of: filling the basin with 100 g of distilled water and assembling the CSES in the insulated enclosure; performing the flux measurement; installing the CSES under the simulator and logging the temperature and mass evolution; and cool down and final mass measurement.

Before installing the CSES under the simulator beam, the flux was measured according to the following procedure. The simulator lamp was ignited and allowed to stabilize for at least 10 minutes. Using a grayscale CMOS camera mounted above the simulator (Supplementary Fig. 17) a sequence of three grayscale images was acquired. First, a thermopile detector (Newport 1918-C) was placed at the focal plane (Supplementary Fig. 18a), and a grayscale image was acquired (Supplementary Fig. 18b) while recording the flux reading from the power meter (Newport 818P-040-55) connected to the thermopile. A correction factor of 0.95 was applied to the power meter reading to account for the difference in thermopile absorptance at the calibration point 1,064 nm vs. the solar weighted absorptance. Second, a diffusely reflecting target (Lambertian target) was placed at the focal plane (Supplementary Fig. 18c), and a grayscale image (Supplementary Fig. 18d) was acquired. The reflected radiance from the Lambertian target is linearly proportional to the incident flux, such that (Supplementary Fig. 18d) represents a relative irradiance map (flux map) at the focal plane. Finally, the CSES and balance were installed at the focal plane (Supplementary Fig. 18e), and a grayscale image (Supplementary Fig. 18f) of the absorber aperture was acquired.

Using the reading from the power meter, the relative flux map (Supplementary Fig. 18d) can be scaled to obtain an absolute flux map. A region-of-interest (ROI) is first traced around the thermopile detector area, as shown in Supplementary Fig. 18b. This same ROI is then superimposed onto Supplementary Fig. 18b. A scaling factor *sf* is then computed according to

$$sf = \dot{q}_{\text{thermopile}} \Big/ \langle \text{GV} \rangle_{\text{ROI}_1} \tag{92}$$

where $\dot{q}_{\text{thermopile}}$ is the average flux over the thermopile as measured by the power meter, and $\langle \text{GV} \rangle_{\text{ROI}_1}$ is the average pixel grayscale value over the thermopile ROI. The scaling factor can then be applied over the whole relative flux map to obtain an absolute fluxmap (Supplementary Fig. 19) according to

$$\dot{q}_{\text{solar}}(x,y) = sf \cdot \text{GV}(x,y) \tag{93}$$

where $\dot{q}_{\text{solar}}(x,y)$ and $\text{GV}(x,y)$ are the absolute flux and grayscale value at pixel coordinate (x,y) respectively. Using the absolute flux map, the average flux over the CSES aperture can be determined. ROI_2 is traced around the absorber area (Supplementary Fig. 18f). This same ROI is then superimposed onto the absolute flux map (Supplementary Fig. 19), from which the total solar input power and average flux can be determined according to

$$\dot{q}_{\text{solar}} = \frac{\int_{\text{ROI}_2} \dot{q}_{\text{solar}}(x,y) dxdy}{\int_{\text{ROI}_2} dxdy} = sf \langle \text{GV} \rangle_{\text{ROI}_2} \tag{94}$$

Image processing was performed in Matlab® R2017b.

In the last step of the flux measurement, the CSES is placed under the solar simulator, marking the start of the experiment. The temperatures and mass were continuously monitored over the course of the experiment. The mass was measured using a balance with a 0.1 mg resolution (A&D EJ3000) connected to the computer. The temperatures were measured using precision fine-gauge K-type thermocouples (Omega 5TC-TT-K-36/40-36/72) connected to the computer through a data acquisition system (Omega DAQPRO-5300). The location of the thermocouples is shown in Supplementary Fig. 7. Due to difficulties in measuring gas temperatures in radiating environments, a custom-built radiation-shielded thermocouple was constructed. The details of the radiation-shielded thermocouple are presented in Supplementary Note 10.

After approximately 1 hour, depending on the flux level, the experiment reached a quasi-steady-state characterized by a constant water reservoir temperature of 100 °C, constant device temperatures, and a near constant evaporation rate. The experiment was run for at least another 2 hours at quasi-steady-state. At the end of the steady-state test period, the lamp was turned off. After turning off the lamp, the device exhibited an initial rapid cooling phase, followed by a slow cooling down to room temperature. In all experiments, it was observed that the evaporation rate stopped within a few minutes of turning off the lamp, supporting our hypothesis that the evaporation is heat transfer limited. After cooling, the mass of the water remaining in the basin was measured.

Supplementary Note 16. Multidimensional conduction model

Multidimensional thermal conduction effects were evaluated using the Finite Element Method (FEM) as implemented in the Solidworks Simulation code. Supplementary Fig. 20a shows a schematic of the model. The CSES device is represented by a block with a prescribed linear temperature gradient (150 °C on the top and 100 °C) on the bottom to mimic conditions under real operation. The top is insulated by the triple-layer transparent polymer glazing system detailed in Supplementary Note 6. The space between the FEP layers was treated as stagnant air. Each surface exposed to the environment was assigned a convective boundary condition with $U_{\mathrm{conv}} = 5\ \mathrm{W\ m^{-2}K^{-1}}$ and $T_{\infty} = 20$ °C. As the purpose of this model was to determine the multidimensional conduction effects, radiation was not included, and is treated separately in the transient model. Temperature dependent thermal conductivity for all materials was taken from manufacturers data.

Supplementary Fig. 20b shows the resulting steady-state temperature distribution through the insulation box. Using the results of the 3D model, the side losses were separated from the top and bottom results using the following methodology. A separate simulation was run where the side insulation was replaced by an adiabatic boundary condition thus forcing one-dimensional conduction in the vertical direction. The heat flux through the top and bottom boundary were then determined from the model. The top and bottom loss heat transfer coefficients were then calculated as

$$U_{\mathrm{top}} = \frac{\dot{Q}_{\mathrm{top}}}{A\Delta T_{\mathrm{top}}} = 1.66\ \mathrm{W\ m^{-2}K^{-1}} \tag{95}$$

$$U_{\mathrm{bot}} = \frac{\dot{Q}_{\mathrm{bot}}}{A\Delta T_{\mathrm{bot}}} = 0.87\ \mathrm{W\ m^{-2}K^{-1}} \tag{96}$$

The resulting heat transfer coefficients were found to be in close agreement with those calculated analytically according to the one-dimensional heat conduction equation accounting for the thermal resistance of each layer in the top and bottom. The side losses were then determined as the difference in the total losses between the 3D and 1D FEA models difference in heat flow rate

$$\dot{Q}_{\mathrm{side}} = \dot{Q}_{\mathrm{3D}} - \dot{Q}_{\mathrm{1D}} \tag{97}$$

and the side loss heat transfer coefficient was then determined as

$$U_{\text{side}} = \frac{\dot{Q}_{\text{side}}}{A\Delta T_{\text{top}}} = 0.96 \text{ W m}^{-2}\text{K}^{-1} \tag{98}$$

Note that there is some arbitrariness in the definition of the side heat loss coefficient, in terms of which temperature difference to use for the normalization. We determined that the best agreement in terms of the overall heat losses was achieved when normalizing by the absorber-ambient temperature difference ΔT_{top}. The side loss heat transfer coefficient was also normalized to the absorber area (15.24 cm × 15.24 cm). The total conduction/convection heat losses are then calculated according to

$$\dot{Q}_{\text{tot}} = U_{\text{top}} A\Delta T_{\text{top}} + U_{\text{bot}} A\Delta T_{\text{bot}} + U_{\text{side}} A\Delta T_{\text{top}} \tag{99}$$

Supplementary Fig. 21 gives a comparison between the component loss model described above and the FEA model, showing good agreement over a wide range of absorber temperatures. The main advantage of splitting the total losses into its components is that the effect of side losses can be explored explicitly. As the system is scaled-up, its aspect ratio can be made large enough such that side losses become negligible and heat flow is essentially one-dimensional.

Supplementary Figures

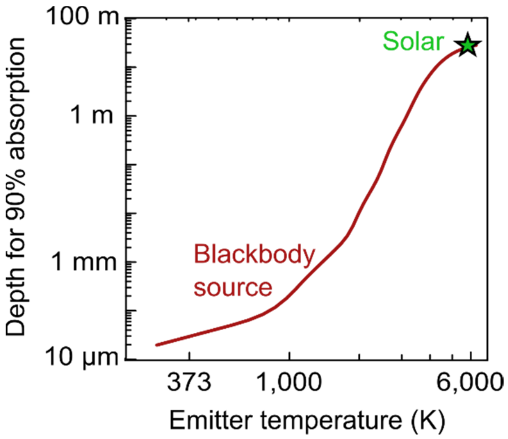

Supplementary Fig. 1 90% photon absorption depth for water. Required depth required to absorb 90% of incident radiation from a blackbody source at different temperatures. The sun behaves like a blackbody at ~6,000 K.

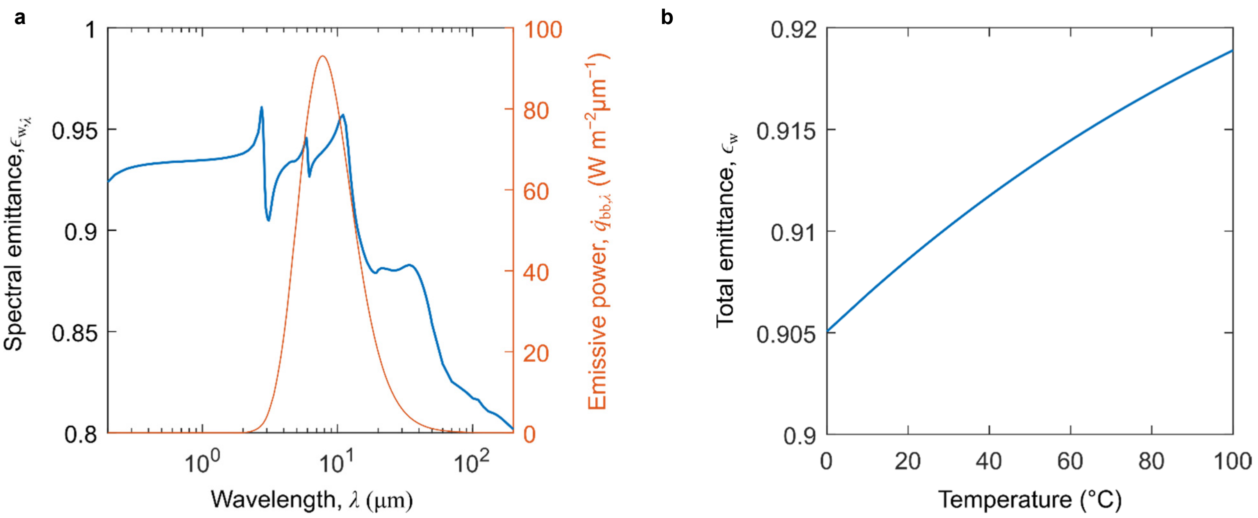

Supplementary Fig. 2 Emittance of an optically thick layer of water. **a** Spectral emittance. Also shown is the spectral emissive power of a blackbody at 100 °C, whose peak coincides with the high-emissivity region of the water spectrum. The low emissivity at wavelength higher than 20 μm is due to increased interface reflectance. The high emissivity in the visible range is due to the assumption of an optically thick medium (which would require many meters of depth) in this region. **b** Total (Planck-averaged) emittance as a function of temperature.

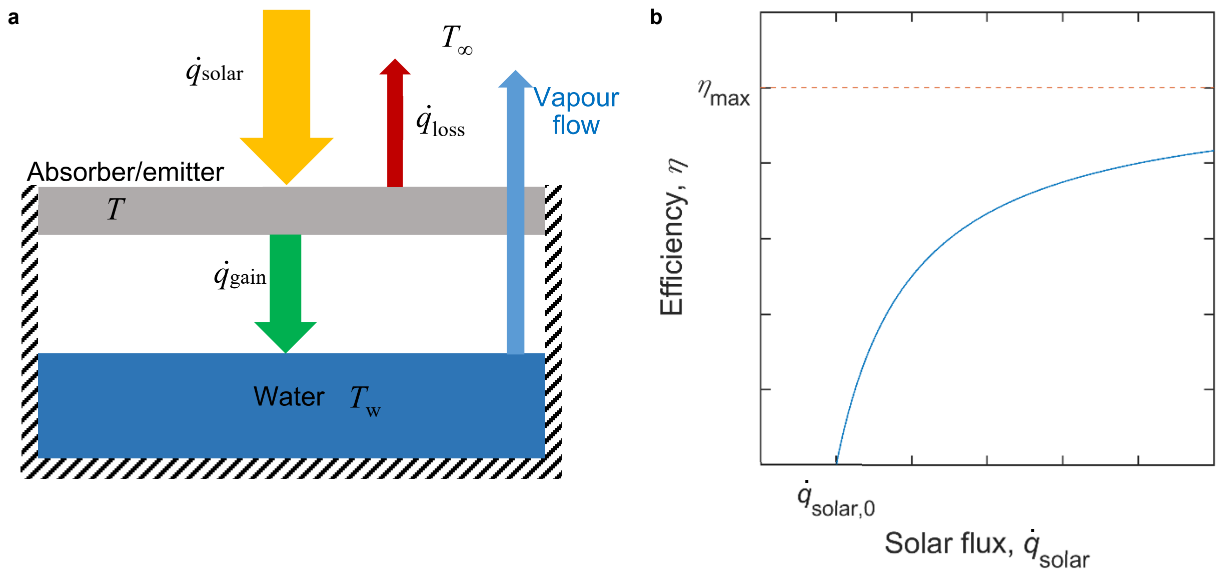

Supplementary Fig. 3 Steady-state analytical heat transfer model of a generic passive solar evaporator. **a** Schematic of energy flows. **b** Efficiency vs. solar flux characteristic curve.

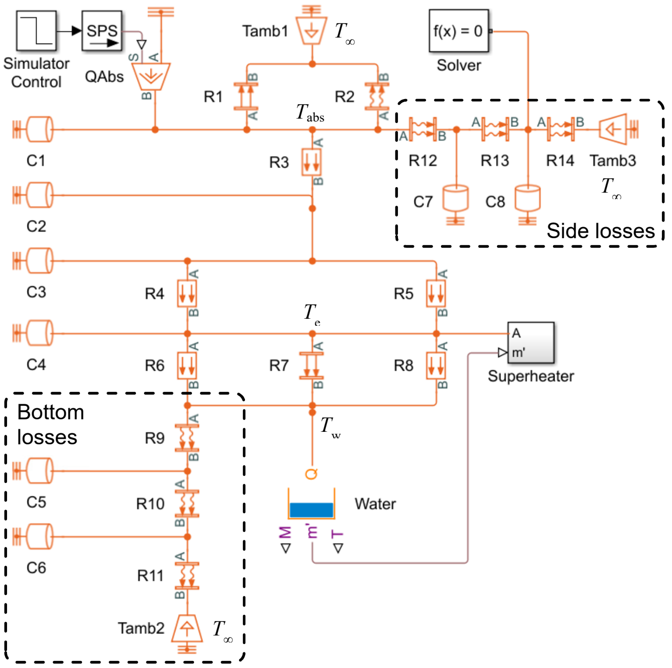

R: Thermal resistance
C: Thermal capacitance
T: Temperature boundary condition
Q: Heat flow rate boundary condition

Tamb1: Ambient temperature
Tamb2: Ambient temperature
Tamb3: Ambient temperature
QAbs: Absorbed solar power, $\dot{Q}_{abs} = \eta_{opt} A \dot{q}_{solar}$

R1: Top radiation losses, $G = 1.55\times10^{-10}$ W/K^4
R2: Top convection losses, $R = 34.44$ K/W
R3: Absorber adhesive layer, $R = 0.11$ K/W
R4: Top gasket conduction, $R = 2.47$ K/W
R5: RVC conduction, $R = 10.11$ K/W
R6: Basin sidewall conduction, $R = 56.37$ K/W
R7: Emitter to water radiation, $G = 8.64\times10^{-10}$ W/K^4
R8: Gas gap convection, $R = 30.58$ K/W
R9-11: Bottom insulation (distributed), $R = 42.21$ K/W
R12-14: Side insulation (distributed), $R = 14.95$ K/W

C1: Selective surface, $C = 19.79$ J/K
C2: Absorber substrate, $C = 55.35$ J/K
C3: Top gasket, $C = 50.30$ J/K
C4: Superheater shell, $C = 129.60$ J/K
C5-6: Bottom insulation (distributed), $C = 153.21$ J/K
C7-8: Side insulation (distributed), $C = 17.02$ J/K

Supplementary Fig. 4 Equivalent circuit diagram representing the transient numerical model.

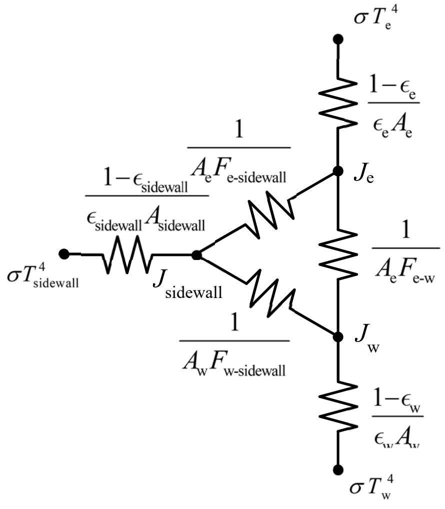

Supplementary Fig. 5 Circuit analogy of the radiative exchange between the emitter, water, and basin sidewalls.

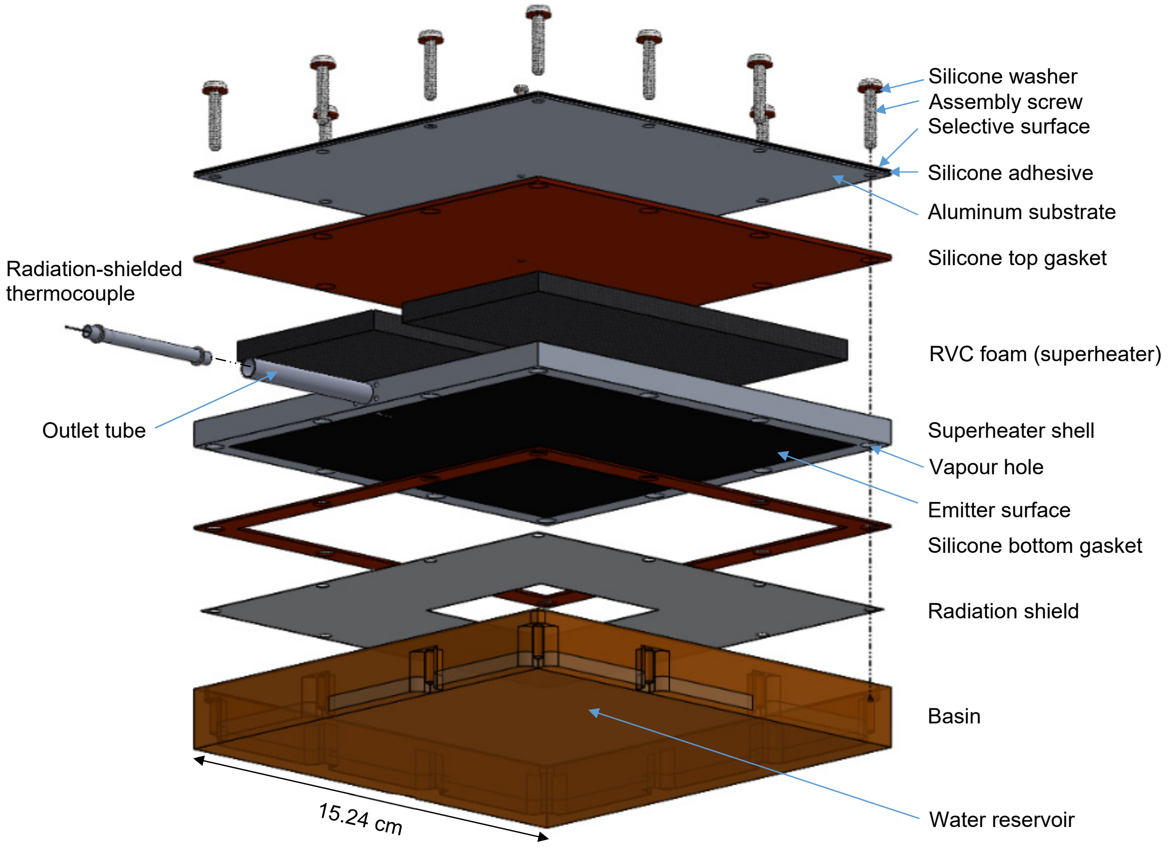

Supplementary Fig. 6 Exploded view of the CSES device showing all main components.

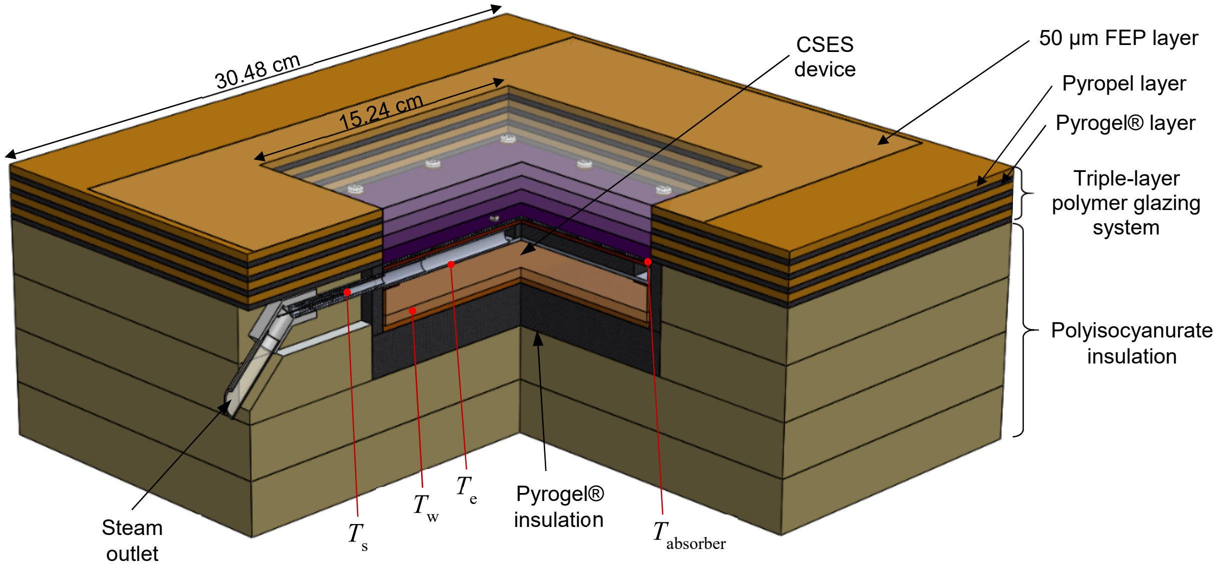

Supplementary Fig. 7 Rendering of the CSES device inside its insulation box and with the triple-layer polymer glazing on top. Also shown are the thermocouple locations used for measured temperatures in the device.

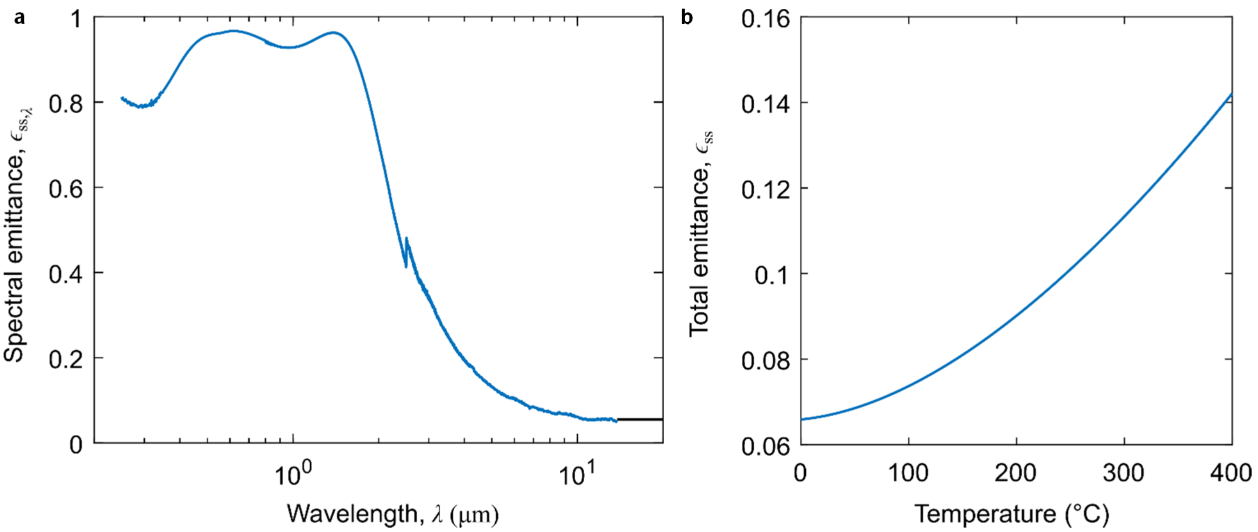

Supplementary Fig. 8 Emittance of selective surface. **a** Spectral emittance computed from 8°/hemi-spherical reflectance measurements. **b** Total (Planck-averaged) emittance as a function of temperature.

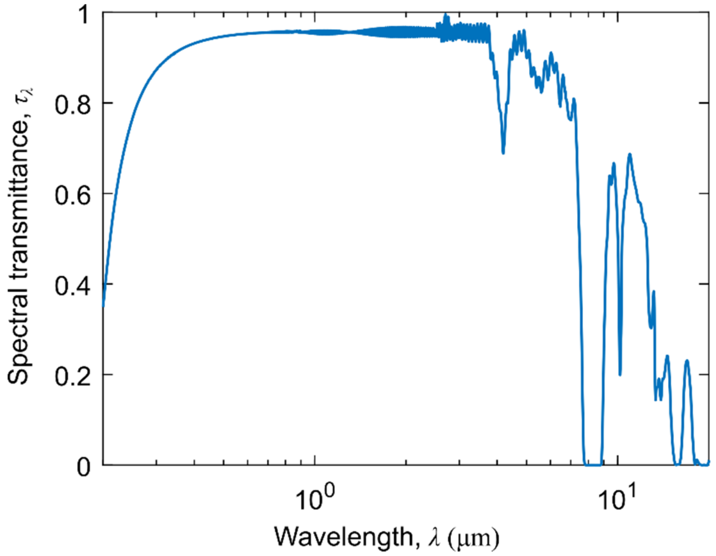

Supplementary Fig. 9 Direct transmission spectrum of 50 μm thick FEP film.

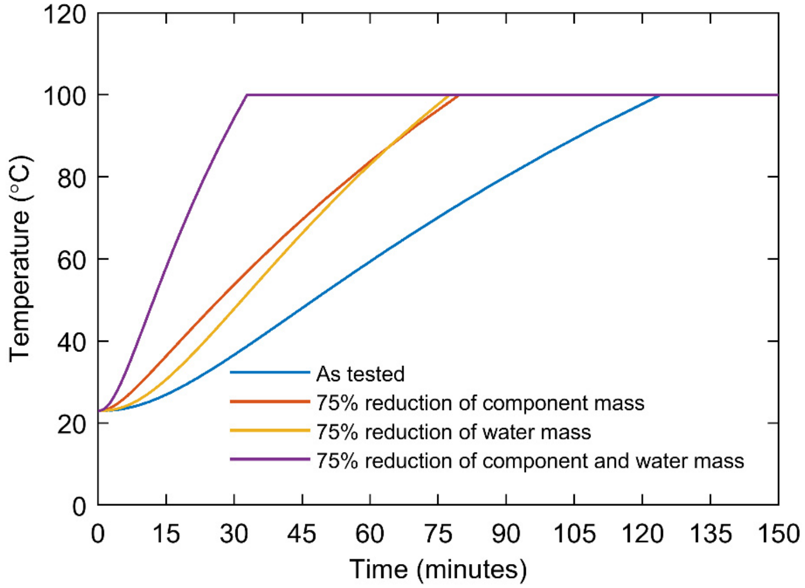

Supplementary Fig. 10 Transient response of the CSES at one sun as predicted by the numerical model. The heat-up time to reach steady state can be reduced significantly by reducing the initial mass (depth) of water in the basin, or by reducing the (thermal) mass of the CSES components, or both.

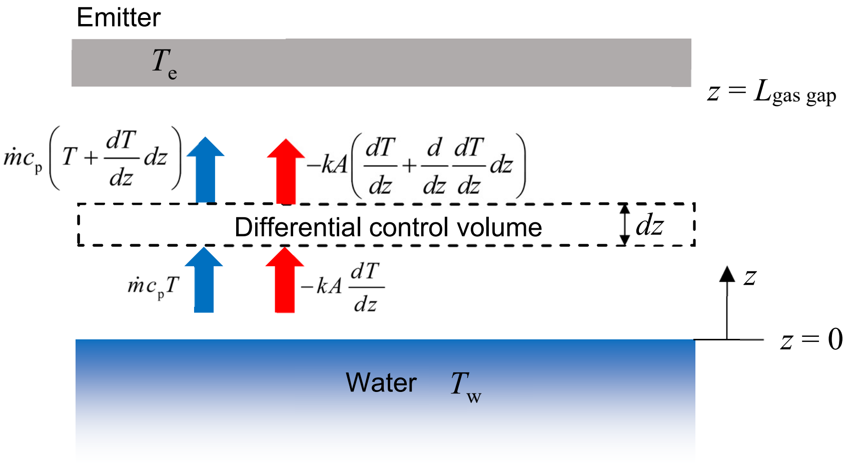

Supplementary Fig. 11 Energy balance on a differential control volume in the gas gap.

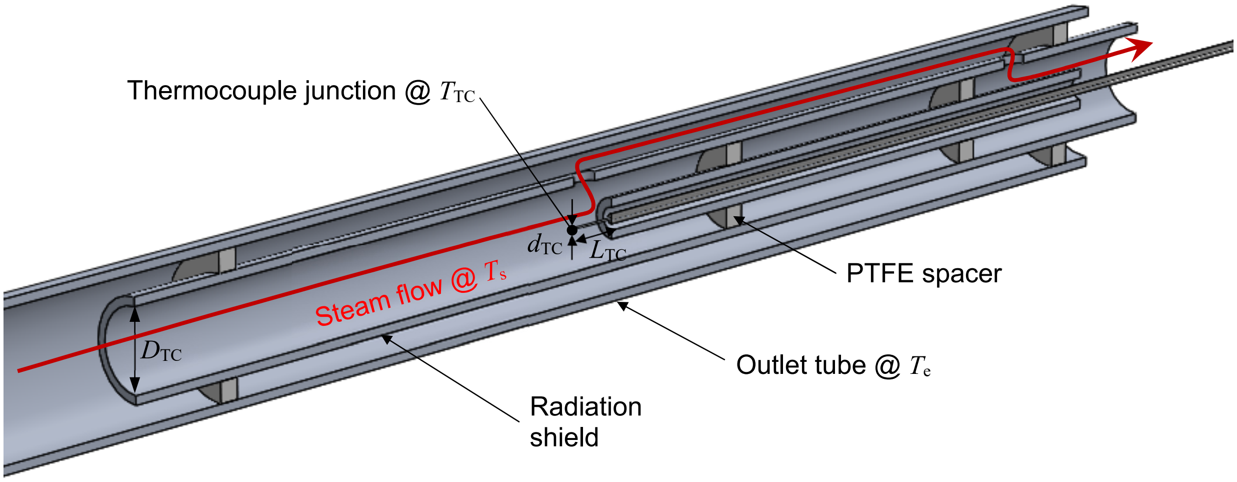

Supplementary Fig. 12 Radiation-shielded thermocouple schematic.

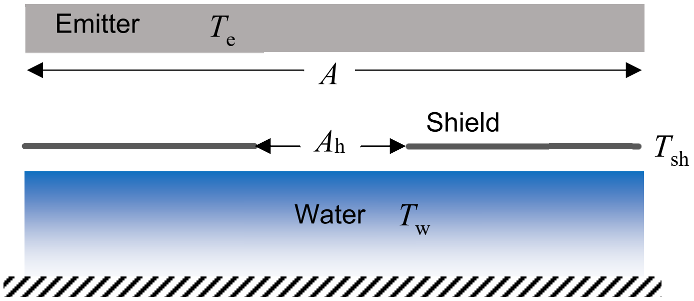

Supplementary Fig. 13 Controlling the degree of superheat via radiative shielding.

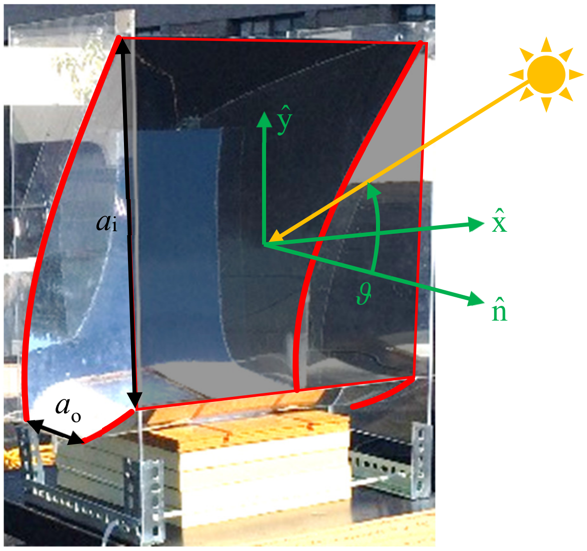

Supplementary Fig. 14 Stationary (non-tracking) asymmetric compound parabolic concentrator (ACPC).

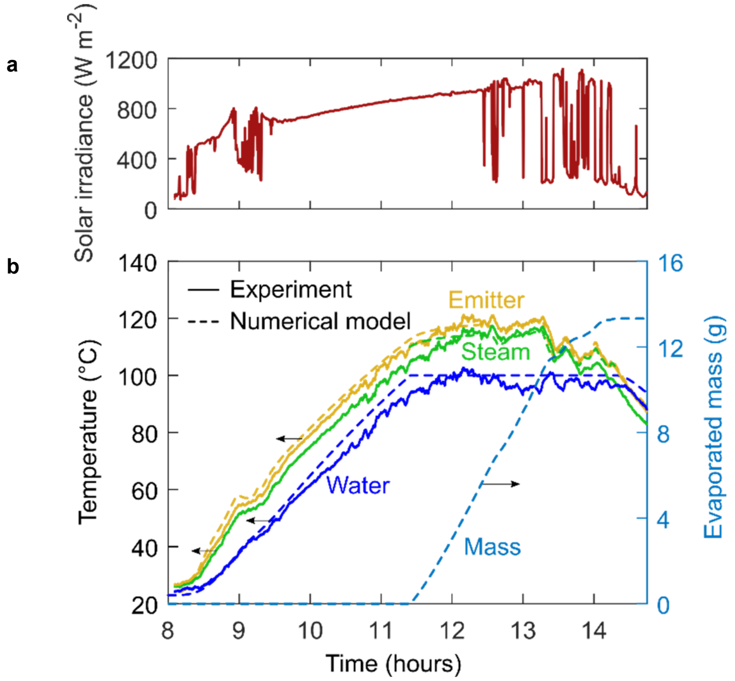

Supplementary Fig. 15 Outdoor operation of the CSES with no concentrator and with vapour condensation and collection. **a** Solar irradiance (global horizontal) measured over the course of the test day, July 13, 2018. **b** Performance of the CSES during the outdoor experiment. Solid lines are measured values and dashed lines are modelled values from the transient model validated vis-à-vis the indoor laboratory experiments.

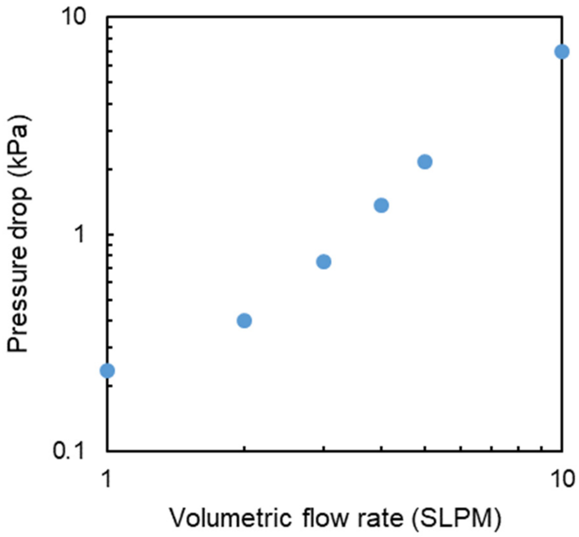

Supplementary Fig. 16 Pressure drop through the system as a function of the volumetric flow rate.

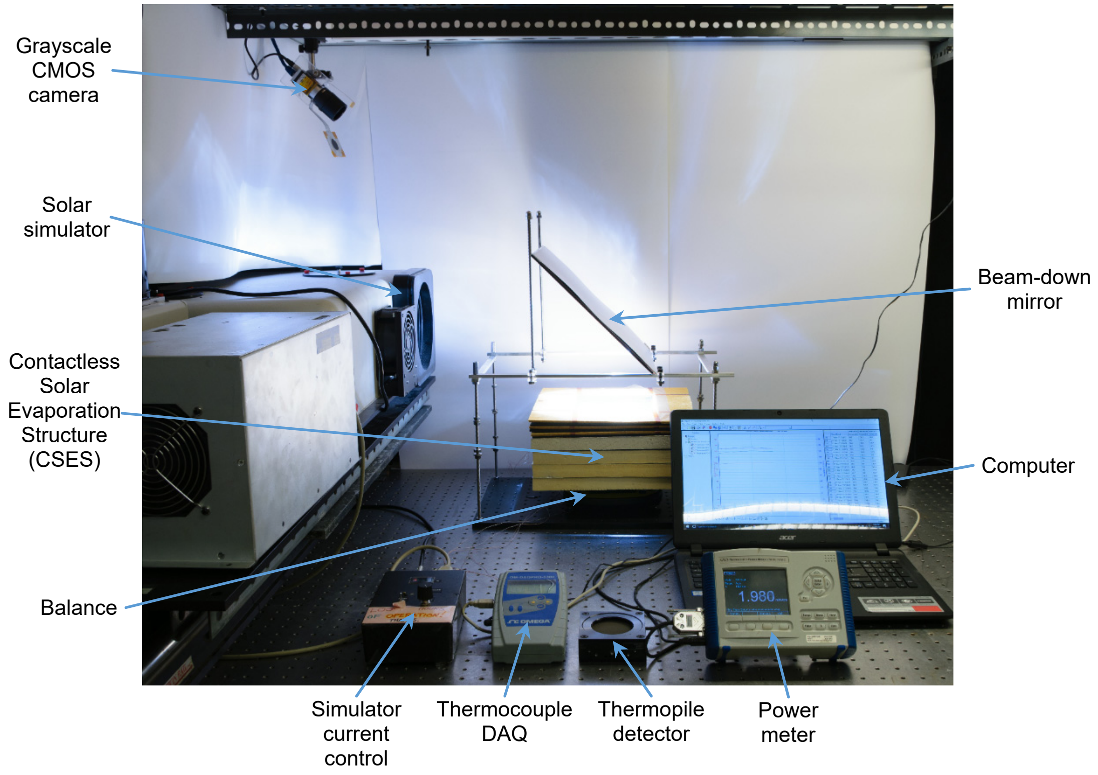

Supplementary Fig. 17 Laboratory experimental setup.

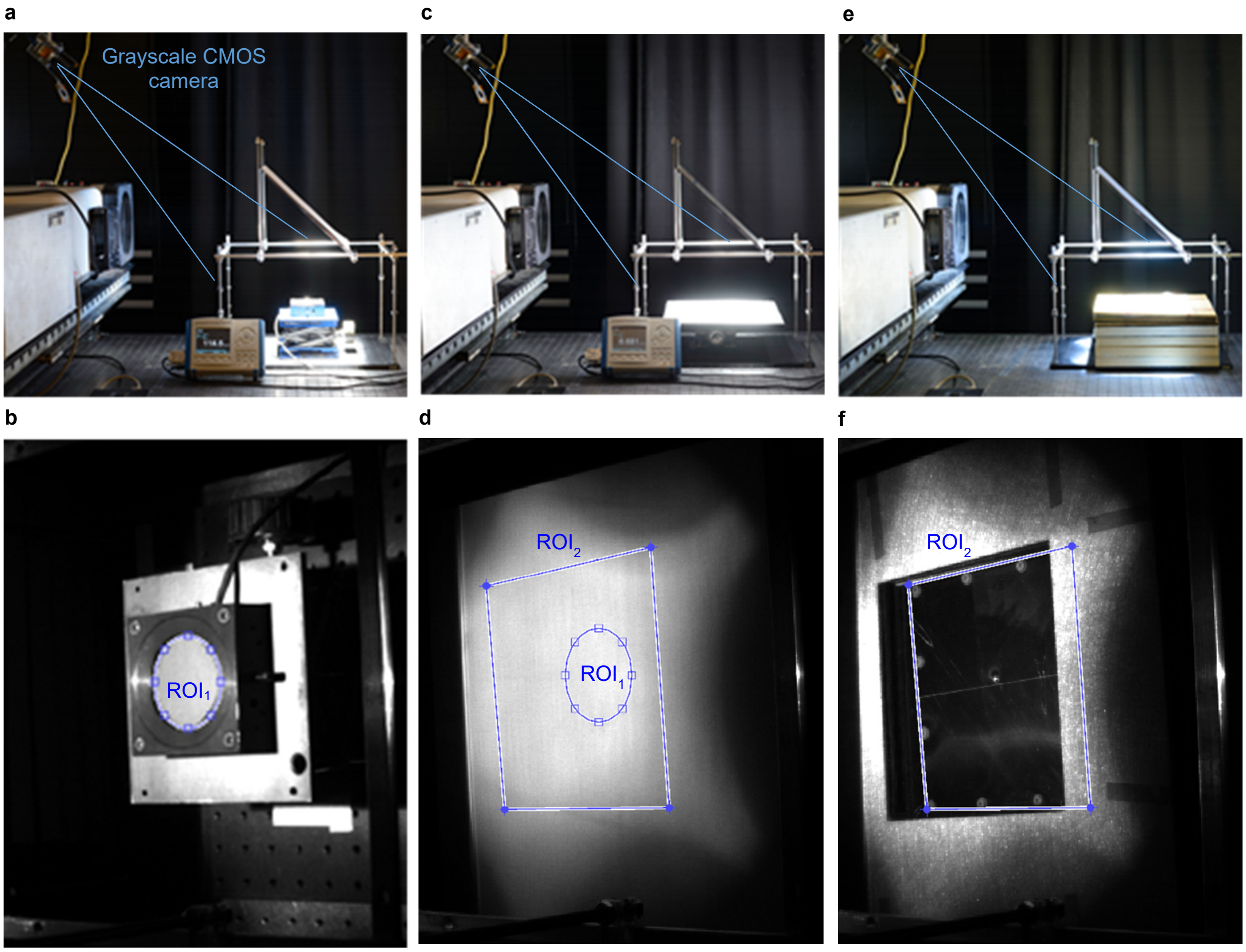

Supplementary Fig. 18 Flux measurement procedure. Before each experiment, a sequence of three images is taken using a grayscale CMOS camera. First, the thermopile detector is placed at the focal plane as in **a**, and a grayscale image **b** of its position is taken as the power meter reading is recorded. Second, a Lambertian target is placed at the focal plane as in **c**, and a grayscale image **d** of it is taken. Finally, the CSES device is installed in place as in **e**, and a grayscale image **f** of the absorber position is taken.

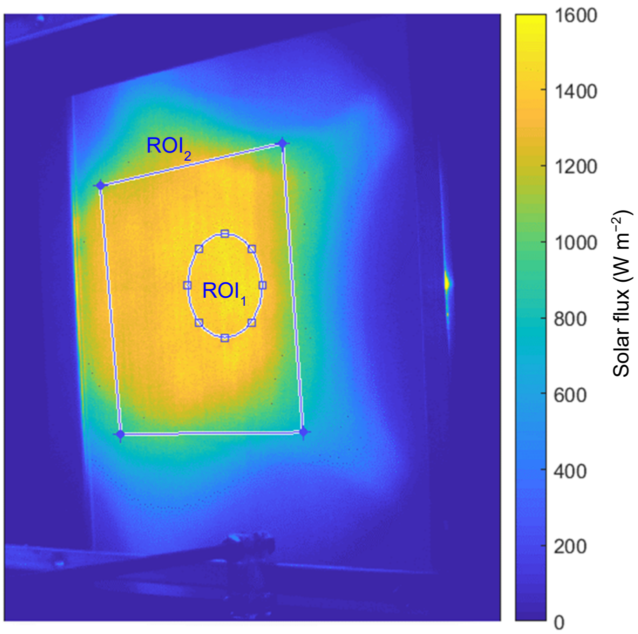

Supplementary Fig. 19 Final absolute flux map. ROI_1 and ROI_2 represent the thermopile detector and absorber locations respectively.

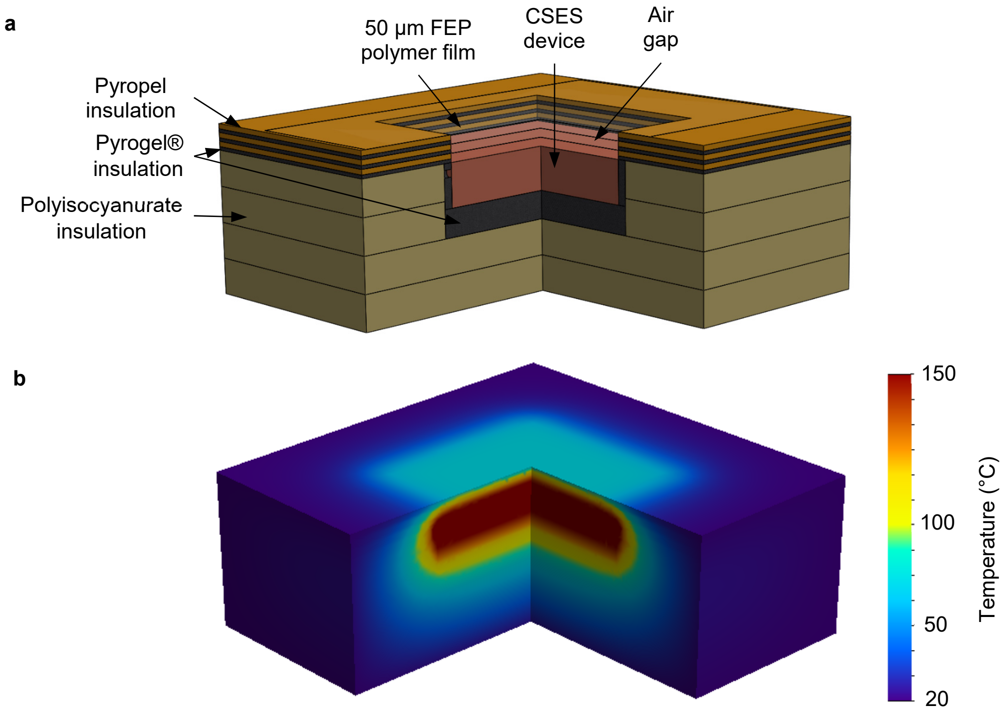

Supplementary Fig. 20 3D steady-state FEM conduction model. **a** Schematic of model. **b** Steady-state temperature distribution through the insulation box when the absorber temperature is 150 °C and the water and basin temperature are 100 °C. Significant lateral temperature gradients are observed, indicating the importance of side losses in the lab-scale setup.

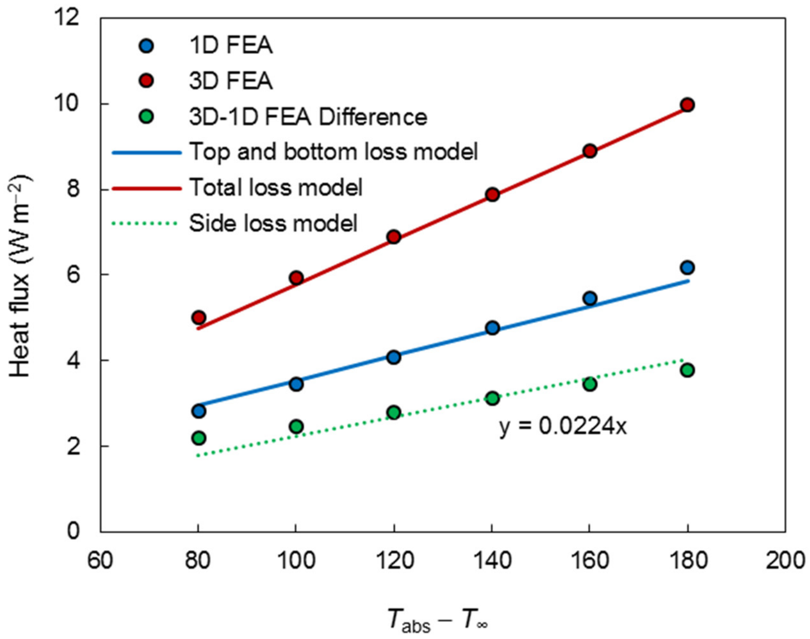

Supplementary Fig. 21 Agreement between the component loss model and the 3D steady-state FEM conduction model.

Supplementary Tables

Supplementary Table 1 Steady-state model parameters for the laboratory scale CSES.

Gain heat transfer coefficient, U_{gain}	12.8 W m^{-2}K^{-1}
Loss heat transfer coefficient, U_{loss}	4.6 W m^{-2}K^{-1}
Superheater effectiveness, ε	0.8
Optical efficiency, η_{opt}	0.758
Break-even flux, $\dot{q}_{solar,0}$	452 W m^{-2}
Maximum efficiency, η_{max}	0.566

Supplementary Table 2 Spectrally-averaged optical properties of the optically important materials used in the device.

**FEP film, 50 µm thick**	
Solar transmittance, τ_{solar}	0.951
Infrared transmittance, τ_{IR}	0.454
**Selective surface**	
Solar absorptance, α_{ss}	0.924
Emittance at 150 °C, ϵ_{ss}	0.081
**Emitter coating**	
Emittance at 150 °C, ϵ_{e}	0.941

Supplementary References

1. Ni, G. *et al.* Steam generation under one sun enabled by a floating structure with thermal concentration. *Nat. Energy* **1,** 16126 (2016).
2. Shen, V. K., Siderius, D. W., Krekelberg, W. P. & Hatch, H. W. *NIST Standard Reference Simulation Website, NIST Standard Reference Database Number 173*. (National Institute of Standards and Technology). doi:10.18434/T4M88Q
3. Çengel, Y. A. *Heat Transfer: A Practical Approach*. (McGraw-Hill, 2003).
4. Shurcliff, W. A. Transmittance and reflection loss of multi-plate planar window of a solar-radiation collector: formulas and tabulations of results for the case of n = 1.5. *Sol. Energy* **16,** 149–154 (1974).
5. Siegel, R. & Howell, J. R. *Thermal radiation heat transfer*. (Taylor & Francis, 2002).
6. Glicksman, L. R. & Leinhard, J. H. V. *Modeling and Approximation in Heat Transfer*. (Cambridge University Press, 2016).
7. Persad, A. H. & Ward, C. A. Expressions for the evaporation and condensation coefficients in the Hertz-Knudsen relation. *Chem. Rev.* **116,** 7727–7767 (2016).
8. Rabl, A. Comparison of solar concentrators. *Sol. Energy* **18,** 93–111 (1976).
9. Orgill, J. F. & Hollands, K. G. T. Correlation equation for hourly diffuse radiation on a horizontal surface. *Sol. Energy* **19,** 357–359 (1977).
10. Reda, I. & Andreas, A. *Solar position algorithm for solar radiation applications, NREL Report No. TP-560-34302*. (NREL, 2003).
11. *ASTM Standard G173-03 - Standard Tables for Reference Solar Spectral Irradiances: Direct Normal and Hemispherical on 37° Tilted Surface*. (ASTM, 2013).
12. Whitaker, S. Forced convection heat transfer correlations for flow in pipes, past flat plates, single. *AIChE J.* **18,** 361–371 (1972).
13. Hawey, W. D. *et al. Measurements of total hemispherical emittance for chromel and for alumel wires, NASA Report TM-X-2359*. (NASA, 1971).
