# Supplementary material for: Contactless steam generation and superheating under one sun illumination
Source: Nat Commun. 2018 Dec 11;9:5086. doi: 10.1038/s41467-018-07494-2 (PMC6290071; doi:10.1038/s41467-018-07494-2)
Supplement: Supplementary file 2 — Description of Additional Supplementary Files [file 41467_2018_7494_MOESM2_ESM.docx]

**Description of Additional Supplementary Files**

**File Name**: Supplementary Movie 1.

**Description**: Operation of the lab-scale contactless evaporation structure. Produced vapour can be seen leaving the outlet tube and rising into the beam of simulated sunlight.
